# Investigating the turbulent dynamics of small-scale surface fires

**DOI:** 10.1038/s41598-022-13226-w

**Published:** 2022-06-22

**Authors:** Ajinkya Desai, Scott Goodrick, Tirtha Banerjee

**Affiliations:** 1 Department of Civil and Environmental Engineering, University of California, Irvine, CA 92697 USA; 2 USDA Forest Service, Southern Research Station, Athens, GA 30602 USA

**Keywords:** Environmental sciences, Natural hazards, Engineering

## Abstract

High frequency (30 Hz) two-dimensional particle image velocimetry data recorded during a field experiment exploring fire spread from point ignition in hand-spread pine needles under calm ambient wind conditions are analysed in this study. In the initial stages, as the flame spreads approximately radially away from the ignition point in the absence of a preferred wind-forcing direction, it entrains cooler ambient air into the warmer fire core, thereby experiencing a dynamic pressure resistance. The fire-front, comprising a flame that is tilted inward, is surrounded by a region of downdraft. Coherent structures describe the initial shape of the fire-front and its response to local wind shifts while also revealing possible fire-spread mechanisms. Vortex tubes originating outside the fire spiral inward and get stretched thinner at the fire-front leading to higher vorticity there. These tubes comprise circulation structures that induce a radially outward velocity close to the fuel bed, which pushes hot gases outward, thereby causing the fire to spread. Moreover, these circulation structures confirm the presence of counter-rotating vortex pairs that are known to be a key mechanism for fire spread. The axis of the vortex tubes changes its orientation alternately towards and away from the surface of the fuel bed, causing the vortex tubes to be kinked. The strong updraft observed at the location of the fire-front could potentially advect and tilt the kinked vortex tube vertically upward leading to fire-whirl formation. As the fire evolves, its perimeter disintegrates in response to flow instabilities to form smaller fire “pockets”. These pockets are confined to certain points in the flow field that remain relatively fixed for a while and resemble the behavior of a chaotic system in the vicinity of an attractor. Increased magnitudes of the turbulent fluxes of horizontal momentum, computed at certain such fixed points along the fire-front, are symptomatic of irregular fire bursts and help contextualize the fire spread. Most importantly, the time-varying transport terms of the turbulent kinetic energy budget equation computed at adjacent fixed points indicate that local fires along the fire-front primarily interact via the horizontal turbulent transport term.

## Introduction

The frequency and severity of wildfires have increased over the last few years and the aggravating global climate (change) presents an increased risk. According to the National Interagency Fire Center^1^, there have been 39108 fires in the year 2021, as of August 7th, 2021 in the USA and the corresponding total burned acreage has increased by 53 % from 2,286,517 acres in 2020 to 3,506,321 acres in 2021. A deeper understanding of wildfire dynamics is an urgent necessity to assist containment operations and fire incident management and prevention. While wildfire modeling has progressed significantly over the past few decades, the progress in terms of observational evidence has been slow. The interaction between the fire and the atmosphere creates a turbulent environment and very limited observations are available to characterize this turbulence as well as the characteristic coherent structures. Measurement of turbulence requires high sampling frequency in time and observations of coherent structures require substantial spatial coverage. Most of the laboratory and field scale observations published in the literature have reported detailed flame structures, which are also important for understanding fire behavior in their own right; while turbulence measurements have been limited to ‘point-in-space’ observations. Being able to measure both, in space and at a high frequency in time, therefore, represents a paradigm shift in our understanding of wildland fire dynamics and fire-atmosphere interaction. In this paper, we will be reporting observations from a particle imaging velocimetry (PIV) experiment that covers a sizeable area in space as well a high sampling frequency, as a flame starting from point ignition spreads. This allows us to track velocity vectors in-situ as the flame structure evolves, as well as surface temperatures, thereby providing unprecedented insights into the complex environment of turbulence in and around a wildfire.

To place this work in context, a brief discussion of the history of wildland fire experiments is warranted. Wildfire researchers have attempted to develop models characterizing wildfire spread since the 1940s. The first empirical formula for the rate of increase of the fire perimeter as a function of wind velocity and fuel moisture content was obtained by Curry and Fons^2^, which later led to the development of the first fire-spread model for heterogeneous fuel beds^3^. Years later, a very simple model for fire growth was obtained^4^ as formulae for the rate of increase in burnt area and fire perimeter per unit time. Anderson^5^ obtained a simple elliptical model for fire propagation through a grassland using Huygen’s principle of wave propagation. Then, Richards^6^ derived a set of first-order, nonlinear differential equations to predict the location of the fire-front at a given time for a point-source-ignition fire. The model was improved in a subsequent study^7^ and a general mathematical model was developed for fire growth in heterogeneous fuel conditions^8^, albeit computationally intensive. These studies provided valuable information regarding the temporal evolution of the fire perimeter (shape and location) based on some parametric shapes but were limited in that regard.

Emerging empirical and semi-empirical models based on laboratory-scale experiments by Rothermel^9^ led to a comprehensive set of useful parametric equations. FARSITE^10^, FlamMap^11^, and BehavePlus^12^ are examples of three computer applications that incorporate this model along with others^13–16^ to produce more extensive results on fire growth. While such models facilitate fire management decisions, they do not account for interaction between the fire and its environment (fire-induced atmospheric turbulence). Several researchers have, therefore, taken to computational fluid dynamics (CFD) models to investigate the detailed flow dynamics underlying wildfire behavior.

WFDS^17,18^ employs the Large Eddy Simulation (LES) method to solve the governing equations for the flow, heat transfer, and chemical processes, and has corroborated several fire spread features observed experimentally. The FIRESTAR system^19^ captured the interactions between surface fires and the surrounding gas flow from simulations in pine stands. Several studies^20–23^ have utilized FIRETEC, which employs a finite-volume solver for its system of governing equations. Alternating regions of upwash and downwash motions^24^ were discovered upwind of the headfire. Streamlines were found to deviate into the flanking direction upwind of the fire-front^22^. Despite their benefits, simulations are limited by the computational overhead and the necessity of simplifying assumptions. The high Reynolds number characterizing the turbulent flow demands higher grid resolution for stability and accuracy. Moreover, fire spread is a result of several simultaneous processes; the Navier Stokes equations are closely coupled with the combustion equations. It was shown in a study^25^ that a 200 s simulation could take upto 6.7 h, while a 3D model^26^ was found to take 3 weeks for 2 min of simulation time in a $1.2\,\text {m}\times 1.2\,\text {m}\times 1.2$ m domain. A Monte Carlo-based wildfire simulation module called WyoFire^27^, which was recently developed for predicting the growth of wildfires in Wyoming, USA, either overestimated or underestimated the wildfire boundaries in grassland fires. Other tools such as QUIC-Fire (a fast running tool that utilizes the phenomenological feature of fire behavior learned from FIRETEC)^28^ are in relatively early stages of development.

With experimental studies in the 1940s focusing mainly on quantifying the spread rate under varied conditions, Fons^3^ acknowledged that understanding the physical processes involved was also necessary to extrapolate the results to more realistic situations. Thereafter, wind-driven fire experiments in shallow fuel beds showed that combustible gases sweep ahead of the head fire-front along the fuel surface indicating that convection, and not just radiation, plays a major role in fire spread^29^. Test fires conducted on large plots of land indicated that fire-whirl formation required the presence of opposing air currents and that ambient flow around the fire resembled flow around a solid object^30^. Through experiments and physical reasoning, Beer^31^ commented on the reliance of the spread rate on atmospheric stability and explained the presence of fire-induced wind in the absence of wind forcing. From several fire experiments conducted in sloping *Pinus halepensis* fuel beds^32^, it was inferred that increased headfire spread rate and stronger whirls for a fire travelling upslope were caused by the increased strength of fire-induced wind behind the fire. Flames in spreading fires are known to intermittently burst forward to ignite fuel particles^33,34^. These are called intermittent bursts or fire bursts. Recent experiments^34^ were able to explain the mechanism behind forward flame-bursts: pairs of counter-rotating vortices that advect hot gases in the direction of headfire propagation.

Although prescribed burns can supply measurements on a management scale, they pose potential risks to expensive equipment and the lives of forest personnel (especially due to wind variability)^35^. This makes small-scale field and laboratory experiments vital to fire-turbulence research. In that regard, particle image velocimetry (PIV) has developed to become a very reliable technique since the term was first used in the literature in the 1980s^36–38^. More accurate and high-resolution (temporal and spatial) measurements of velocity vectors in two-dimensional (2D) flow fields have reduced much conjecturing around wall-bounded turbulent flow and have provided more detailed structural pictures^38^. Although the technique has been used to study flow features in premixed laminar flames^39^, the effect of puffing in pool fires^40^, to obtain air-entrainment rates in pool fires^41^, etc., it has only recently begun to be availed to study fire spread in vegetative fuels^42^. Despite the challenges due to variability in external conditions and flow seeding, PIV measurements^42^ in a 10 m $\times ~5$ m bed of excelsior provided useful insights on the response of the flame dynamics to wind conditions. The presence of a “plume-dominated” region followed by “wind-dominated” region^43^ was observed. In the plume-dominated region, flames act like a barrier to the wind and there is an influx of fresh air from the downwind side of the fire. In the wind-dominated region, winds accelerate near the flame region. Flow crosses the fire-front to push hot gaseous products onto unburnt fuel. In a successive study^44^, PIV data was collected at a sampling frequency of 10 Hz in a 0.71 m $\times ~0.71$ m domain to study a fire propagating upslope. They concluded that a higher acquisition rate (higher sampling frequency) would facilitate a better visualization of the velocity fluctuations and fluid dynamic structures as the fire progresses in time.

The current manuscript attempts to highlight some of the features of the turbulent flow during a grassland fire using PIV data sampled at a high frequency of 30 Hz in the horizontal plane (top view). A point source of ignition has been used in this experiment, in which pine needles were spread by hand to mimic natural needle cast in field situations. In this work, we first report observations regarding how the ambient environment responds to the presence of a flame that spreads from point ignition. Next, we seek to explore some of the coherent structures that characterize the flow to obtain insights on the mechanism of fire spread in the absence of a preferential ambient wind-forcing direction. Another important question that we address is how fire flames at different locations along the fire-front interact with each other. The paper is organized as follows. We begin with preliminary observations on the fire growth with the help of snapshots of the local wind velocity, streamlines, and surface temperature. The analysis is expanded further: quantities derived from the velocity data, such as cross-correlation contours, vorticity vectors, and turbulent momentum fluxes, are computed for insights into the coherent structures that characterize the fire-induced turbulent flow. Estimates of the time, length, and velocity scales at play, as well as the rate of spread (RoS) are obtained next. We then explore the role of the individual terms of the turbulent kinetic energy (TKE) budget equation in the evolution of the fire. Finally, the contribution of this work is summarized along with directions for future work.

## Results

### Flow field and streamlines

**Figure 1:**
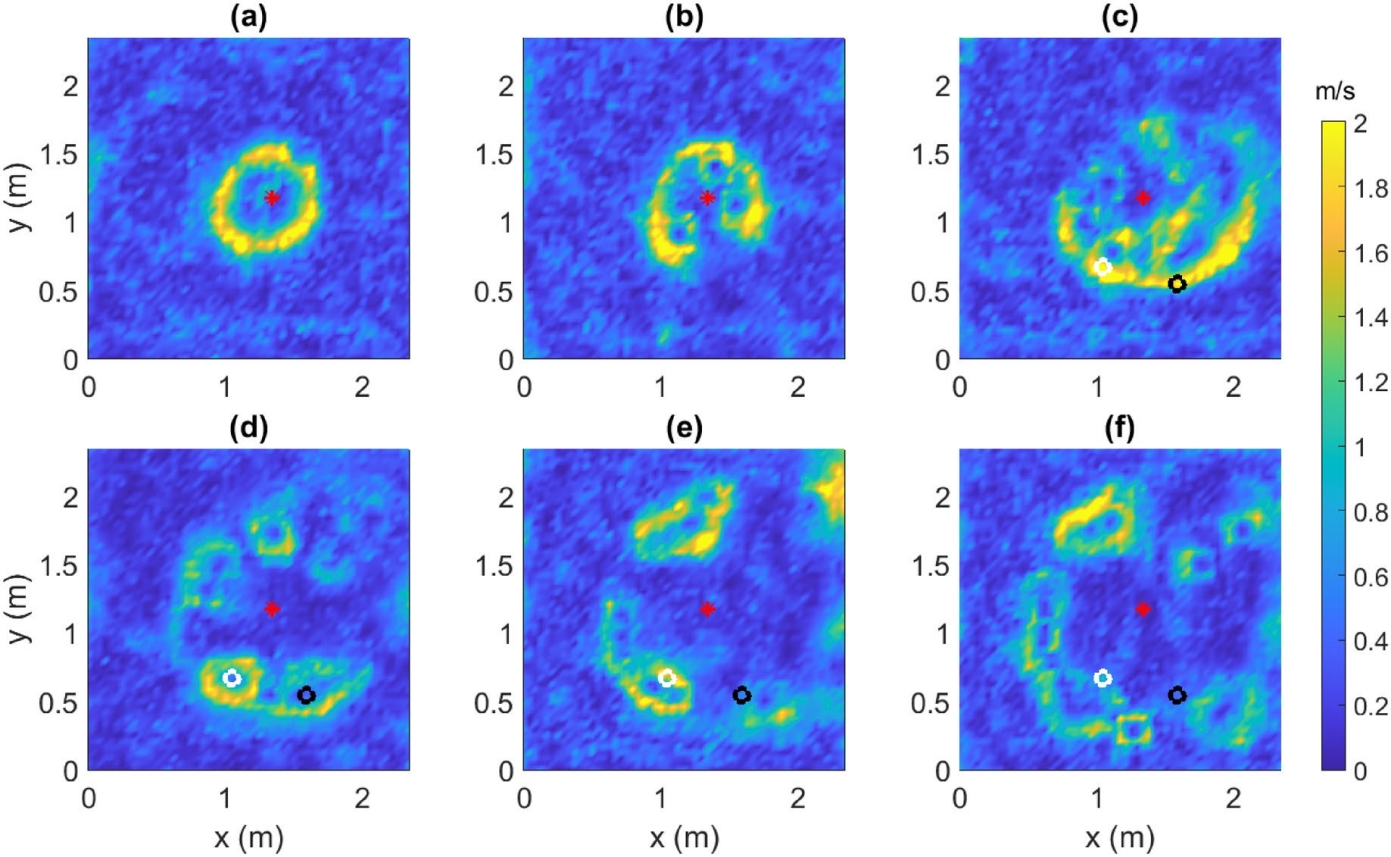
The magnitude of horizontal velocity in the domain at time $t=$ (**a**) 100 s, (**b**) 110 s, (**c**) 175 s, (**d**) 247.5 s, (**e**) 300 s, and (**f**) 350 s. The red asterisk indicates the ignition point (IP). The white and black circles in (**c**–**f**) indicate the coordinates of the fixed points formed around $t=247.5$ s in (**d**). [Generated using MATLAB R2021a].

Figure 1 shows color contours of the magnitude of the horizontal velocity ($u_H = \sqrt{u^2 + v^2}$) of the local flow in the presence of the fire at different instances of time (*t*) elapsed since ignition. It must be noted that the fire-front cannot be clearly delineated from the contours of $u_H$ alone. Nevertheless, it can be argued that the fire-front is on the inner side of the region of large $u_H$ (yellow), since the fire creates a low-pressure warmer core that draws in cooler air from the surrounding region. It can be seen that up to $t=110$ s, the fire-front is relatively stable and defined by a closed elliptical or circular shape (Fig. 1a). Around $t=110$ s, it can be seen that local instabilities begin to set in and the fire starts to cease being a closed curve and forms small “pockets” of fire (Fig. 1b). These “pockets” are centred at relatively fixed points, i.e. points of extremely low local horizontal wind velocity. After residing at these fixed points for several seconds, the fire “pockets” travel to a neighboring location, drawing in air from the surrounding region of unburnt fuel. As the fire grows, neighboring “pockets” coalesce to form larger ones (Fig. 1e–f). These snapshots provide a clear validation of Huygen’s principle of wave propagation, cited in earlier works^5,6,8^ as a model for fire growth. According to the Huygen’s principle, every point on the fire perimeter at any time *t* behaves as an ignition point for a local fire; the envelope of the local fires at time $t+\text {d}t$ determines the new fire-front at that time. An interesting question arises regarding whether there is some form of energy exchange between two such adjacent local fires, which shall be explored later. Two fixed points that are formed at time $t=247.5$ s (Fig. 1d) and remain relatively fixed for several seconds thereafter are depicted using a white circle ($x = 1.05$ m, $y= 0.67$ m) and a black circle ($x=1.59$ m, $y=0.54$ m) in Fig. 1c–f. Henceforth, these two fixed points are referred to as FP1 and FP2, respectively, for brevity. The time-varying signals at these points shall be used for our analysis on turbulent momentum fluxes and the TKE budget.

**Figure 2:**
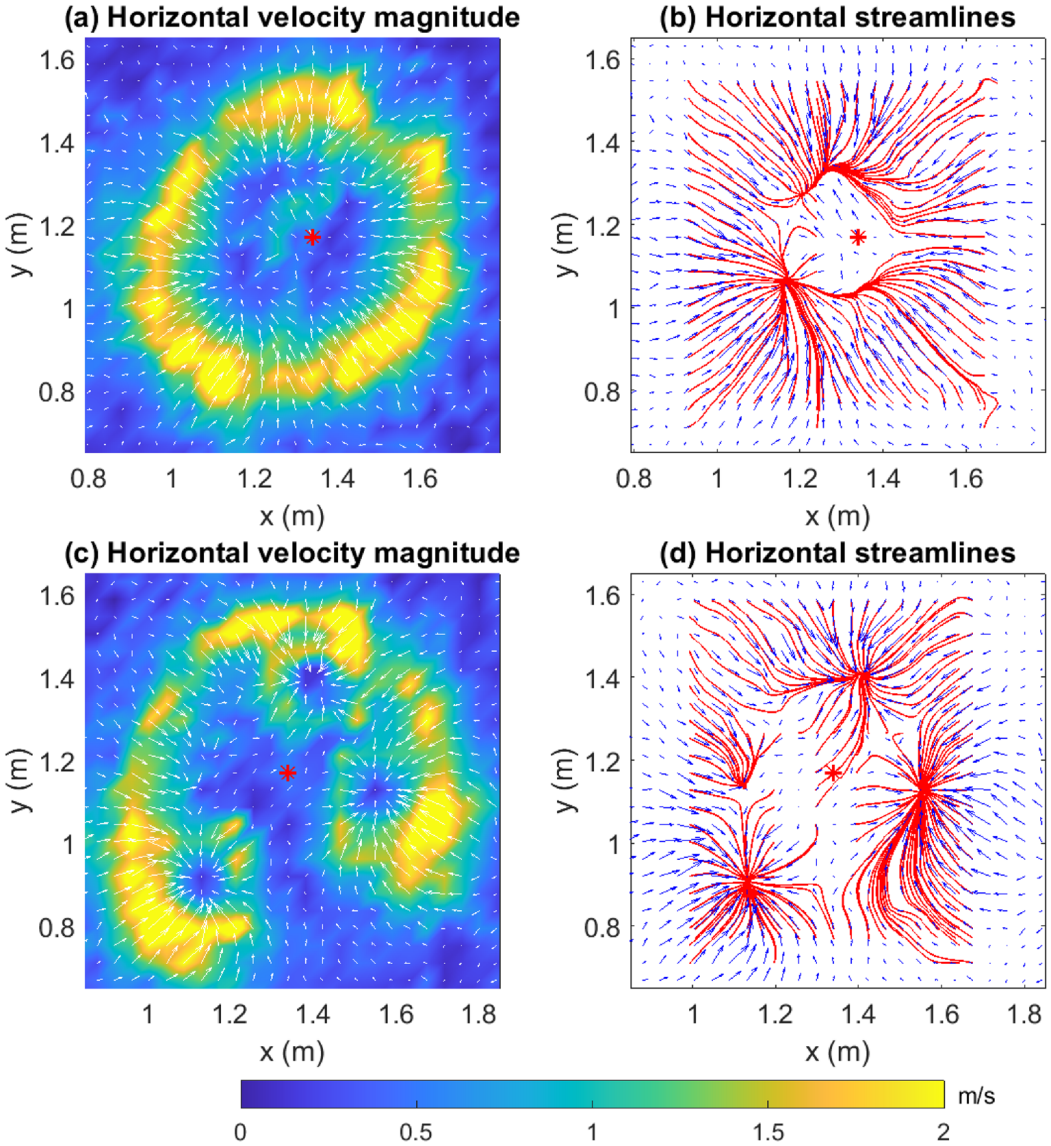
Horizontal velocity ($\mathbf {u}_H$) vectors overlaid on color contours of $u_H$ at $t=$ (**a**) 100 s and (**c**) 110 s along with streamlines overlaid on horizontal velocity ($\mathbf {u}_H$) vectors at $t=$ (**b**) 100 s and (**d**) 110 s. The red asterisk indicates the IP. Arrows have been scaled by 1.5. [Generated using MATLAB R2021a].

**Figure 3:**
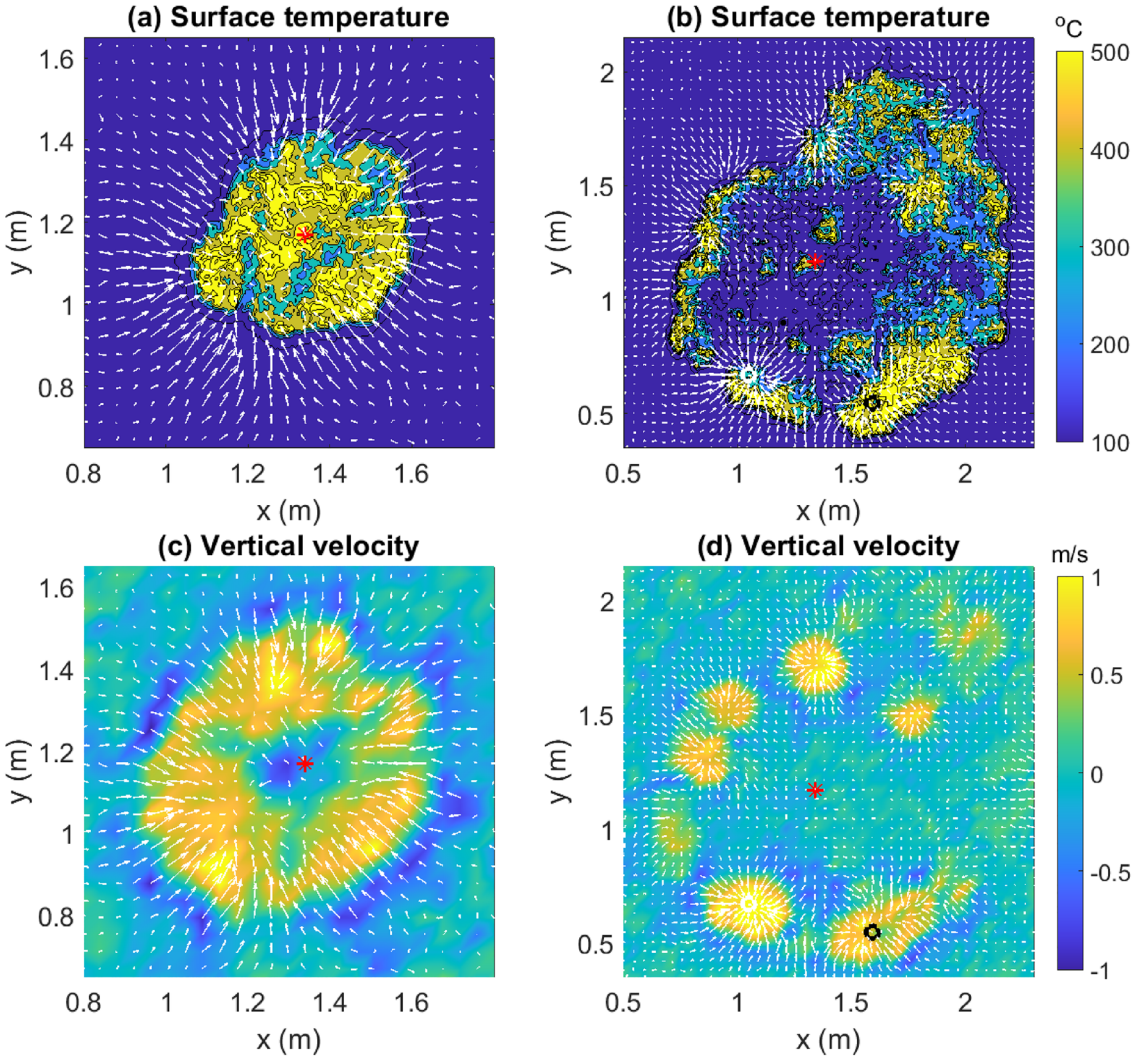
Color contours of the surface temperature ($T_s$) with overlaid vectors of horizontal velocity ($\mathbf {u}_H$) at $t=$ (**a**) 100 s and (**b**) 247 s, demonstrating the entrainment of colder ambient air. Color contours of vertical velocity (*w*) and vectors of horizontal velocity ($\mathbf {u}_H$) at $t=$ (**a**) 100 s and (**b**) 247.5 s, demonstrating the influx of colder ambient air from the region of downdraft. Arrows have been scaled by 1.5. Red asterisk indicates the IP. White and black circles represent FP1 and FP2, respectively. [Generated using MATLAB R2021a].

For the sake of brevity, we shall refer to the part of the fuel-field burnt by the fire at a certain instant of time as the “inside” of the fire and the unburnt region as the “outside” of the fire. The color contours of $u_H$ have been overlaid with vectors for the horizontal velocity ($\mathbf {u}_H=u\hat{\mathbf{i }} + v\hat{\mathbf{j }}$) alongside the corresponding streamlines in Fig. 2. The $\mathbf {u}_H$ vectors (Fig. 2a,c) indicate that the relatively still and potentially colder (as corroborated later by the surface temperature contours) air from the “outside” is drawn into the warmer “inside” of the fire. The influx of air (and thus oxygen) contributes to the combustion process and helps in sustaining the fire. However, this fire-induced local wind also appears to exert a dynamic pressure upon the fire-front, providing resistance to the fire spread rather than accelerating it. A similar observation was made by Canfield et al.^22^ for wind-driven fire, wherein a low-pressure region was found to form on the downwind side of the fire-front. Moreover, Fig. 2b shows that streamlines entering the fire get curved away from the fire-core. These streamlines meet near the inner edge of the fire-front and appear to shield it from the innermost core of the fire. The curving of the streamlines is indicative of a dynamic pressure differential between the inner and outer edge of the fire front^22^. Additionally, the divergence of the streamlines indicate that the flow decelerates at the fire front, as also evidenced by the size of the velocity vectors. The fact that the streamlines terminate indicates that they leave the horizontal plane where the streamlines are computed. In this case, the opposing wind vectors join to form an updraft region^22^ as indicated later in Fig. 3. Unlike observations made in some previous works that studied the behavior of a fire driven by the wind in a particular direction^22^, the streamlines do not appear to drive the fire-front forward to contribute to the fire spread. Furthermore, it can be observed that when fire-pockets begin to form at time $t=110$ s (Fig. 2d), the streamlines entering these pockets converge at the fixed points enclosed by them.

**Figure 4:**
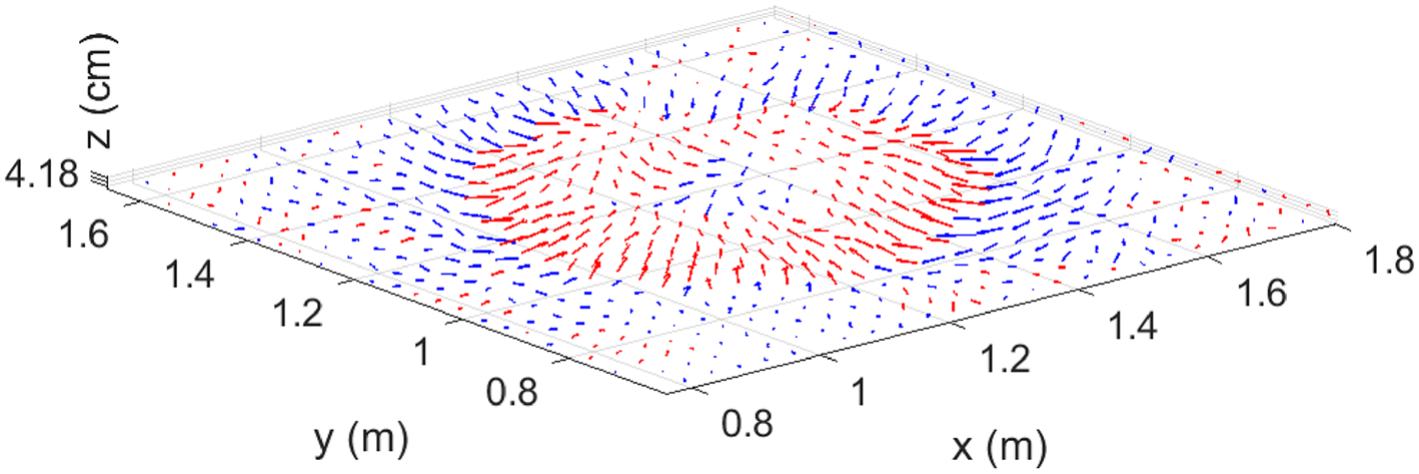
An isoparametric view of the total velocity ($\mathbf {u} = u\hat{\mathbf{i }} + v\hat{\mathbf{j }} + w\hat{\mathbf{k }}$) vectors plotted 4.18 cm above the surface of the fuel bed and zoomed into the active combustion region of the domain at $t=100$ s. Red arrows indicate updrafts ($w>0$), while blue arrows indicate downdrafts ($w<0$). [Generated using MATLAB R2021a].

Color contours of the surface temperature ($T_s$) are shown in Fig. 3a,b at $t=100$ s and $t=247$ s, respectively, along with overlaid vectors of horizontal velocity ($\mathbf {u}_H$). The darkest blue regions are cooler ambient regions, while the yellow regions are high temperature regions that also include regions of active flaming. The fire-front can be delineated from the edge of the more brightly colored contours before they merge into the darkest blue (ambient temperature) regions. Although measurements of air temperature are unavailable, it is reasonable to assume that they would be lower above cooler surfaces (darkest blue regions). Thus, the influx of (cooler) ambient air into the fire-core (Fig. 3a) cools the fire-core as the fire-front expands. In some cases (Fig. 3b), contours of the highest temperature lie “outside” the fire-front. This is because the fire heats the unburnt fuel on the outside via radiation. At ignition temperature, this fuel begins to burn, causing the fire to spread outward. Furthermore, we present color contours of the vertical velocity (*w*) with overlaid vectors of the horizontal velocity ($\mathbf {u}_H$) in Fig. 3c–d. Dark blue regions are downdraft regions, while yellow regions are those of updraft. We expect the fire-core to manifest as the region of updraft surrounded by the annular region of downdraft. This allows a clear demarcation of the fire-front. Moreover, the cooler ambient in-drafts over the downdraft region decelerate as they pass through the updraft region. The fire-front at $t=247.5$ s, as shown in Fig. 3d, affords an interesting observation. The pockets of fire at each fixed point are regions of updraft, which are separated by regions of downdraft. The envelope of the fire-perimeter of each of these pockets constitutes the fire-front, which consequentially comprises alternating regions of upwash and downwash verifying similar observations made in the literature^24,34^.

Finally, a three-dimensional flow picture is constructed from an isoparametric view of the net flow ($\mathbf {u} = u\hat{\mathbf{i }} + v\hat{\mathbf{j }} + w\hat{\mathbf{k }}$) at $t=100$ s as shown in Fig. 4. It is observed that the net velocity vectors at the fire-front are inward and upward (red arrows). We, therefore, expect the flame to be tilted inward as is characteristic of small or point-source fires that advance against the wind (in this case, fire-induced wind)^45^. On the “outside” of the fire-front, the net velocity vector is inward and downward (blue arrows) indicating the presence of a downdraft region.

### Coherent structures

According to Jiménez^46^, coherent structures in wall-bounded turbulent flows can be defined as structures “with enough internal dynamics to behave relatively autonomously from any remaining incoherent part of the flow.” Indeed, coherent structures serve to provide a direction to the seemingly random nature of the flow and their study constitutes an integral part of the analysis that makes turbulent flow during fires less mysterious. In this section, correlation contours and vorticity vectors/rollers are explored for a clearer picture of the fire structure and evolution. The eddy fluxes of momentum at the selected fixed points (FP1 and FP2) have also been explored for signatures of fire bursts (defined in the introduction).

#### Cross correlation contours

The two-point spatial correlation at time *t* is defined as follows^47^:

1 $$\begin{aligned} r_{ij}(x,y,t) = \frac{\overline{u_i(x_0,y_0,t) u_j(x,y,t)}}{\overline{u_i'^2(x_0,y_0)}^\frac{1}{2}~\overline{u_j'^2(x,y)}^\frac{1}{2}} \end{aligned}$$

**Figure 5:**
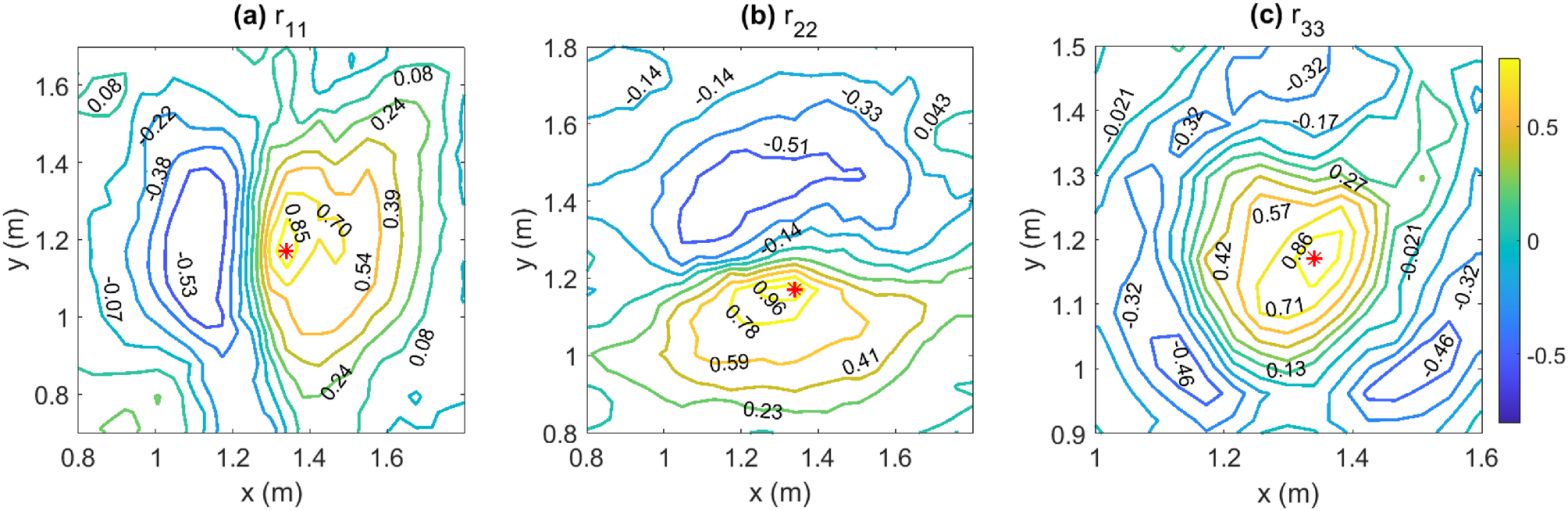
Cross-correlation contours of (**a**) *u* velocity ($r_{11}$), (**b**) *v* velocity ($r_{22}$), and (**c**) vertical velocity ($r_{33}$) plotted close to the IP (red asterisk). [Generated using MATLAB R2021a].

In Eq. (1), ($x_0,~y_0$) represents the IP ($x_0 = 1.34\,\text {m},~ y_0=1.17\,\text {m}$). Coefficients of cross-correlation with the IP are evaluated across the entire domain. Figure 5 presents contours of $r_{11},~ r_{22},~\text {and}~r_{33}$ and the IP is plotted using a red asterisk.

The *y*-directionally (north-south) elongated structures of the $r_{11}$ contours (Fig. 5a) indicate a *y*-directional uniformity in the *u* (east-west) velocity. For the first 100 s of ignition, the *u* velocity component remains negative ($u<0$) at the IP (not shown here). Hence, the positively correlated contours on the east of the IP represent *westward* *u* velocity ($u<0$), while the negatively correlated contours on the west of the IP represent *eastward* velocity ($u>0$). Similarly, the *x*-directionally elongated structures of $r_{22}$ contours (Fig. 5b) indicate *x*-directional uniformity in the *v* velocity. Again, for the first 100 s of ignition, the *v* velocity remains positive ($v>0$) at the IP (not shown here). The positively correlated contours on the south of the IP represent northward *v* velocity ($v>0$), while the negatively correlated contours on the north of the IP represent southward *v* velocity ($v<0$). We interpret the cross-correlation contours as a measure of the retainment of flow memory (recorded at the IP, in this case) across space. The *y*-directionally elongated $r_{11}$ contours are indicative of the entrainment of ambient air from the eastern and western sides of the domain as a relatively quick and bulk response to ignition, the earliest sign of the presence of fire. The *x*-directionally elongated $r_{22}$ contours are similarly indicative of the entrainment of ambient air from the northern and southern sides of the domain as a similar (quick and bulk) response.

Elliptical structures with decreasing values of $r_{33}$, where $r_{33}>0$, can be observed with increasing distance from the IP in Fig. 5c. These are interpreted as follows. Since the IP is initially a region of updraft, contours that are positively correlated with the IP are inferred to be regions of updraft. Beyond these structures, we observe contours that correlate negatively with the IP and do not encompass it. These can be interpreted as regions of downdraft beyond the fire perimeter. A shift in the direction of fire propagation towards the north-west can also be observed from the contours. This can be attributed to a shift in the direction of the local wind or heterogeneity in the fuel bed in the vicinity of the IP. Thus, contours of $r_{33}$ give a clear picture of the evolution of the fire-front during the first minute or so.

#### Vorticity and rollers

**Figure 6:**
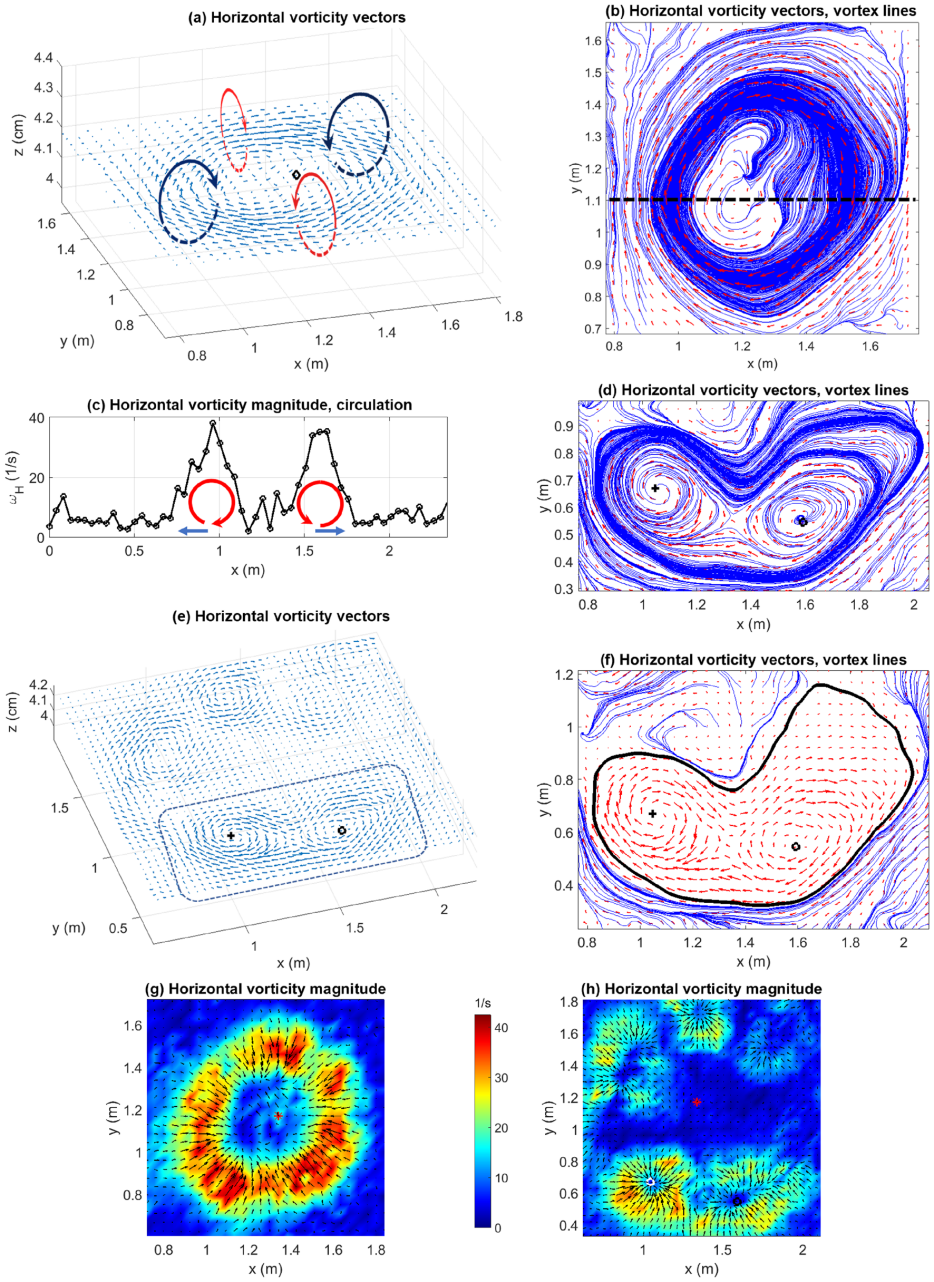
Horizontal vorticity vectors at $t=$ (**a**) 100 s (dashed curved arrows represent vortices) and (**e**) 247.5 s (dashed rectangle highlights the region around FP1 and FP2), and vortex lines for $t=$ (**b**) 100 s (black dashed line represents $y=1.1$ m) and (**d**) 247.5 s (zoomed in around FP1 and FP2). (**c**) Magnitude of $\varvec{\omega }_H$ plotted along with a schematic diagram of the circulation in the vertical plane as indicated by the vortex tubes in (**b**), at $y=1.1$ m. (**f**) Vortex lines (thick black solid) isolating the vorticity field around the fixed points. Color contours of the magnitude of $\varvec{\omega }_H$ with overlaid horizontal velocity ($\mathbf {u}_H$) vectors (black arrows, scaled by 1.5) at $t=$ (**g**) 100 s and (**h**) 247.5 s. Red asterisk represents the IP. [MS PowerPoint 365 used to generate arrows in (**a**) and (**c**); all panels generated using MATLAB R2021a].

It has been reported in the literature^34^ that fire-induced counter-rotating vortices participate in fire spread by advecting hot gases towards fuel particles, causing ignition. From this experiment, it is possible to compute the vorticity as the curl of the velocity field ($\omega =\nabla \times \mathbf {u}$) and examine its spatial structure. In this section, we first look at horizontal vorticity ($\varvec{\omega }_H = \omega _x\hat{\mathbf{i }} + \omega _y\hat{\mathbf{j }}$) vectors in the domain both before and after the fire-front disintegrates. The effect of the vertical component of vorticity ($\varvec{\omega }_z$) on the vortical structures is also analyzed.

Figure 6a depicts $\varvec{\omega }_H$ for $t=100$ s. It should be noted that the vorticity here is induced by both buoyant convection and shear. The fire-induced turbulent fluctuations induce the formation of vortices as shown in Fig. 6a,c. A simple right-hand-thumb rule can be used to determine the direction of circulation of the vortices (curved arrows that go in and out of the plane in Fig. 6a). It is important to note that the vectors plotted in Fig. 6a,e) are not velocity vectors but vorticity vectors in the horizontal plane. The hand-drawn dashed curved arrows (Fig. 6a) show the actual circulations that result in these vorticity vectors given by $\varvec{\omega }_H = \omega _x\hat{\mathbf{i }} + \omega _y\hat{\mathbf{j }}$. Moreover, these circulation structures confirm the presence of counter-rotating vortex pairs on opposite sides reported elsewhere as a key mechanism for fire spread^34^. Note that while Finney et al.^34^ hypothesized the existence of these vortex pairs from images of laboratory fires, the present experiment enables us to quantify these vortices for the first time, which represents a significant advancement in the field.

Vortex lines are lines whose tangents are parallel to the local vorticity vector. As seen from Fig. 6b, vortex lines originating on the outside of the fire spiral inward into the fire-front where the vorticity enhances significantly and then into the fire core where the vorticity eventually dissipates. The vortex lines drawn through each point of a closed curve constitute the surface of a vortex tube. The spatial density of these lines increases at the fire front indicating that vortex tubes get stretched thinner at that location, before dispersing again at the fire core. To conserve circulation, when a vortex tube is stretched (thereby making it thinner), the magnitude of the vorticity has to increase, which is also confirmed by Fig. 6g–h. A schematic of the vertical cross-section of the vortex tubes at $y=1.1$ m is shown in Fig. 6c. If we imagine the fire-front to be located at the regions of highest vorticity magnitude, we can see how the vortices (red curved arrows) provide an outward push to hot gases that are close to the surface of the fuel bed (blue horizontal arrows). The advection of hot gases onto the unburnt fuel would result in ignition, causing the fire to spread “outward”. An example of a post-instability vortical pattern is shown in Fig. 6e. Figure 6e shows the horizontal vorticity vectors for $t=247.5$ s with a focus on the vorticity surrounding FP1 and FP2 (black cross and black circle, respectively). The vortical pattern around a fire pocket centred at a fixed point mimics the pre-instability vortical pattern around the IP at $t=100$ s (Fig. 6a). Moreover, the vortices that surround FP1 are also seen to interact with the vortices that surround FP2. This indicates a possible mechanism for interaction between two such neighboring flames. Furthermore, Fig. 6d shows that the vortex lines that originate from within the fire-pocket around FP1 spiral outward and away. These vortex lines spiral inward and into the fire-pocket around FP2. The thick black solid line in Fig. 6f indicates the presence of a vortex line that separates the fixed points from the rest of the vorticity field, secluding the vortical interactions between the two fixed points from the rest of the vorticity field.

Color contours of the magnitude of the horizontal vorticity ($\varvec{\omega }_H$) have been shown in Fig. 6g–h along with overlaid arrows representing the horizontal velocity ($\mathbf {u}_H$) vectors. For both, $t=100$ s (Fig. 6g) and $t=247.5$ s (Fig. 6h), regions of high magnitude of $\varvec{\omega }_H$ correspond to regions of higher $\mathbf {u}_H$ (longer arrows). This demonstrates a strong correlation between the strength of the vortices and the air entrained by the flame. This is an important observation in the context of fire-whirl formation discussed later.

**Figure 7:**
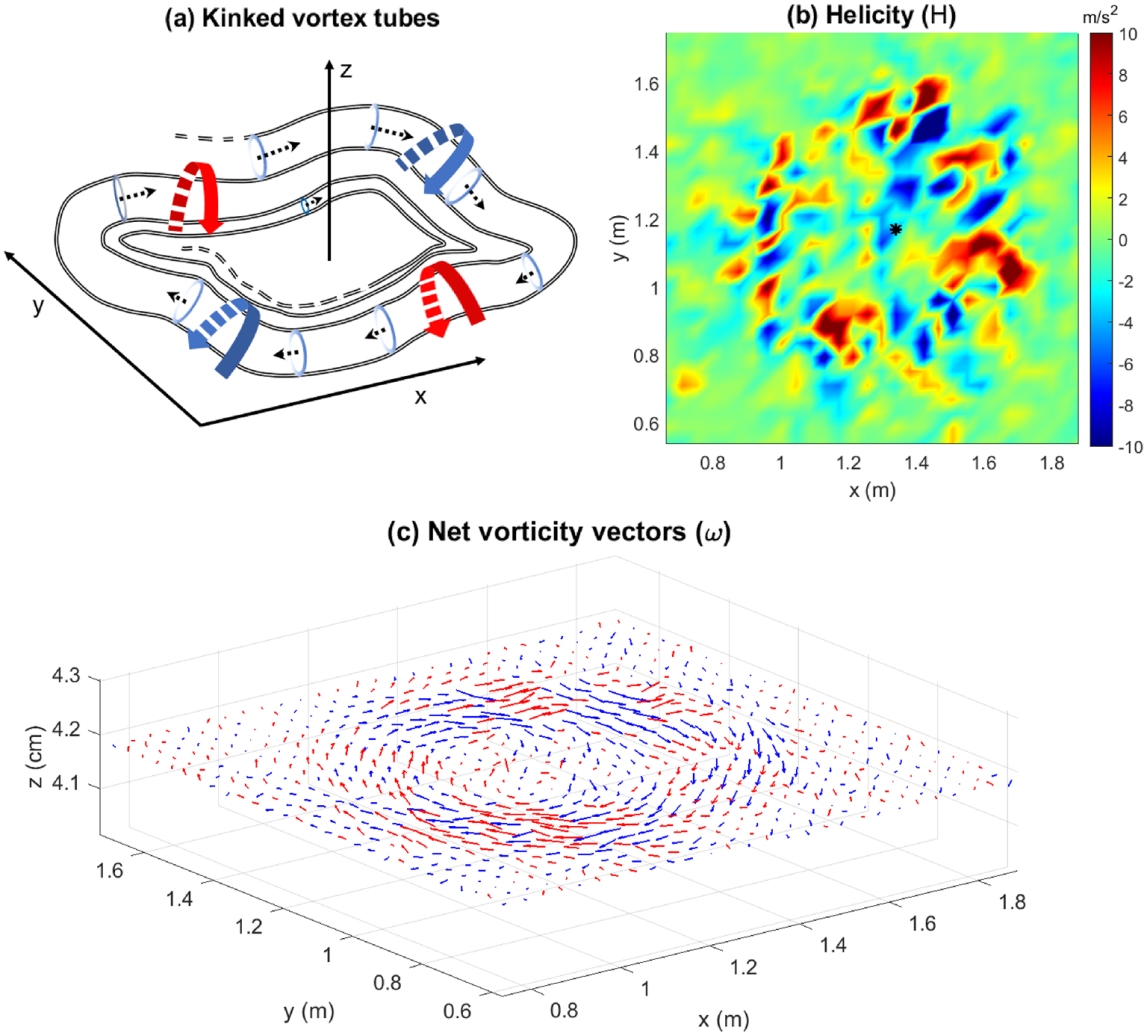
(**a**) A schematic diagram showing the kinked vortex tubes (not to scale), (**b**) helicity (*H*), and (**c**) net vorticity vectors ($\varvec{\omega } = \omega _x\hat{\mathbf{i }} +\omega _y\hat{\mathbf{j }}+\omega _z\hat{\mathbf{k }}$) demonstrating that eddies precess alternately away from (red arrows) and toward (blue arrows) the surface at $t=100$ s. Pairs of counter-rotating vortices are shown in (**a**). Black asterisk in (**b**) represents the IP. [MS PowerPoint 365 used to create (**a**); (**b**) and (**c**) generated using MATLAB R2021a].

Another feature of the vortices can be observed from the net vorticity vector ($\varvec{\omega }$), i.e. from the addition of the vertical component of the vorticity to $\varvec{\omega }_H$. Vectors of the net vorticity ($\varvec{\omega }$) at $t=100$ s, shown in Fig. 7c, indicate that eddies making up the vortex tubes described above precess upward (red arrows) and downward (blue arrows), alternately. This causes kinking of the resulting vortex tubes (schematic diagram in Fig. 7a). This feature is better visualized with the help of color contours of helicity. The helicity (*H*) at any point in the flow field is given by $H = \mathbf {u}\cdot \nabla \times \mathbf {u} = \mathbf {u}\cdot \varvec{\omega }$, where “$\cdot$” represents taking the inner product. The sign of the helicity is indicative of the relative angle between $\mathbf {u}$ and $\varvec{\omega }$: $H>0$ implies that the angle is acute, while $H<0$ indicates that the angle is obtuse. Color contours of the helicity are shown in Fig. 7b, wherein alternating regions of positive helicity (red) and negative helicity (blue) are observed along the fire-front. Since the fire-front comprises updrafts (red arrows in Fig. 4), an acute angle with $\mathbf {u}$ indicates that $\varvec{\omega }$ is pointed away from the surface (red arrows in Fig. 7c) and an obtuse angle with $\mathbf {u}$ indicates that $\varvec{\omega }$ is pointed towards the surface (blue arrows in Fig. 7c). This suggests that the eddies precess upward and downward alternately, resulting in the kinking of the vortex tubes along the fire-front.

We take the opportunity to discuss the possibility of fire-whirl formation here. It is known that essential conditions for fire-whirl formation include the presence of a vorticity-generation mechanism and entrainment of air to the generated vortex column via a radial boundary layer^48^. Both conditions are satisfied in the current study. It is known from Fig. 2b that the streamlines penetrating the fire-front curve away from the fire core testifying to an active $\varvec{\omega }_z$ component. The vertical component of flow velocity in the vicinity of the fire-front (Fig. 4) would be able to direct the curving streamlines and advect (or tilt) the vorticity rollers (observed in Fig. 7a) upward, away from the surface, potentially resulting in a fire whirl. A more complete exploration of fire whirls is anticipated in a future study, with three-dimensional data collection.

#### Eddy fluxes of horizontal momentum

**Figure 8:**
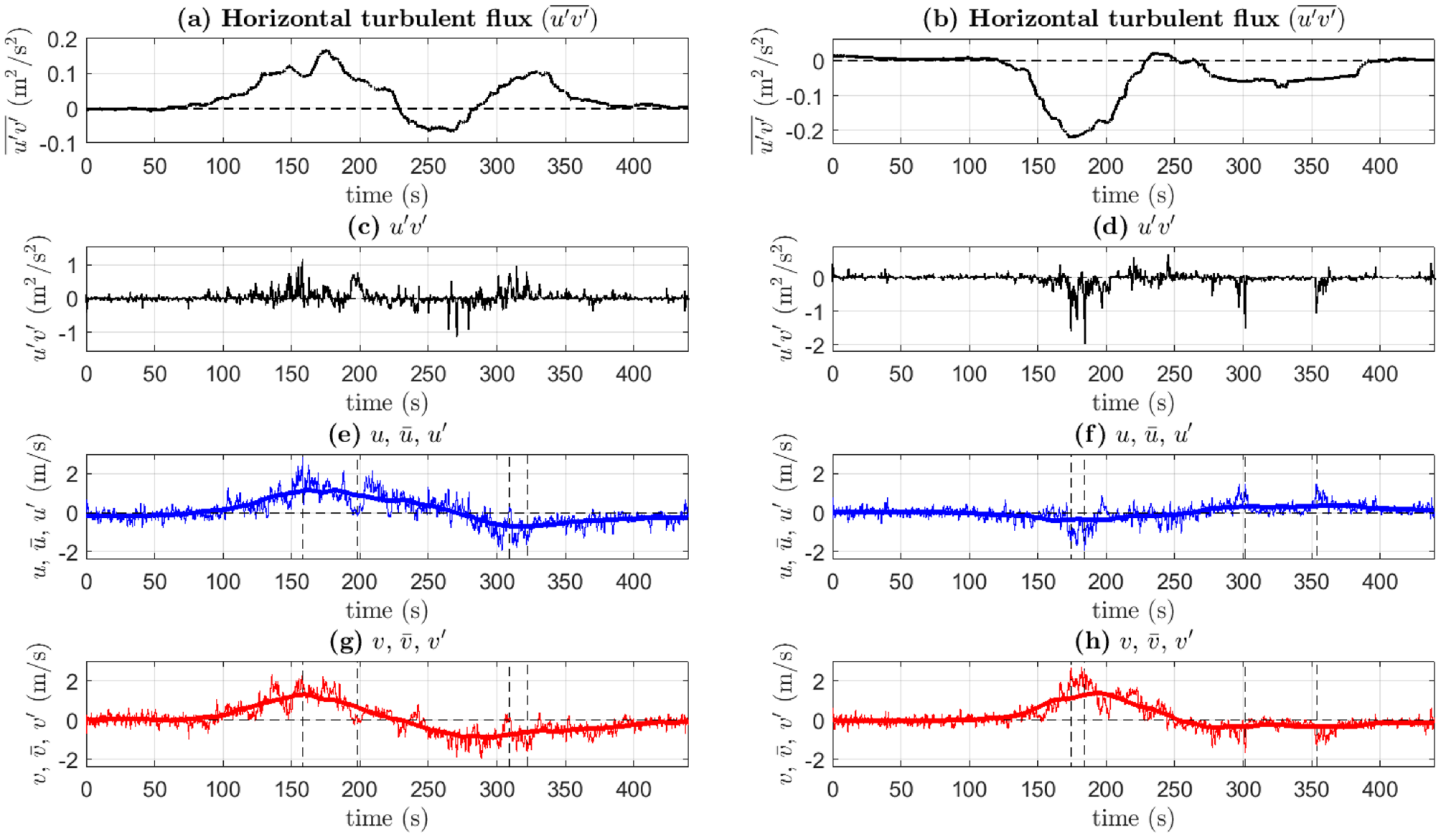
(**a**) The horizontal turbulent flux ($\overline{u'v'}$), (c) $u'v'$, (e) *u*, $\overline{u}$ (thick solid line), and $u'$, and (**g**) *v*, $\overline{v}$ (thick solid line), and $v'$ at FP1. (**b**) The horizontal turbulent flux ($\overline{u'v'}$), (**d**) $u'v'$, (f) *u*, $\overline{u}$ (thick solid line), and $u'$, and (**h**) *v*, $\overline{v}$ (thick solid line), and $v'$ at FP2. Dashed vertical lines in (**e**) and (**g**) represent $t=$ 158 s, 198 s, 309 s, and 322 s. Dashed vertical lines in (**f**) and (**h**) represent $t=$ 174 s, 184 s, 301 s, and 353 s. [Generated using MATLAB R2021a].

Turbulent momentum fluxes (Reynolds stresses normalized by fluid density) facilitate a redistribution of momentum in the flow via eddies. In this section, we analyze the horizontal momentum flux ($\overline{u'v'}$) and the product of the horizontal turbulent fluctuations ($u'v'$) at the selected fixed points (FP1 and FP2) (Fig. 8). Let us first focus on FP1 (white circle in Fig. 1d). An increase in the magnitude of $\overline{u'v'}$ indicates the presence of the fire at or in the vicinity of this point (Fig. 8a). However, the 1-min moving averages do not provide information regarding what the individual peaks in $u'v'$ represent. Consider the peaks in $u'v'$ at $t=158$ s and $t=322$ s (Fig. 8c). At $t=158$ s, the mean *x*-directional flow is eastward ($\overline{u}>0$, Fig. 8e), while the mean *y*-directional flow is northward ($\overline{v}>0$, Fig. 8g). From Fig. 1c–d, it can be seen that at this location, $u'<0$ and $v'<0$ would assist in propagating the fire away from the IP (south/west/southwest-ward), while $u'>0$ and $v'>0$ would combine to create the opposite effect. Therefore, $u'(>0)$ and $v'(>0)$ at $t=158$ s (Fig. 8e,g) interact to impede the spread of the fire away from the IP at this location; this is seen as a peak in Fig. 8c. A similar phenomenon is also observed at $t=309$ s when the mean flow supports the fire spread away from the IP ($\overline{u},~\overline{v}<0$), while the fluctuations resist it ($u',~v'>0$). At $t=322$ s, the mean *x*-directional flow is westward ($\overline{u}<0$, Fig. 8e), while the mean *y*-directional flow is southward ($\overline{v}<0$, Fig. 8g). The fluctuations $u'(<0)$ and $v'(<0)$ at $t=322$ s (Fig. 8e,g)) interact to accelerate the mean flow and drive the fire south-west with sudden force away from the IP, causing a fire-burst. This phenomenon is also observed at $t=300$ s, while burst-like peaks are also observed at $t=148$ s, $t=198$ s (Fig. 8c).

At FP2 (black circle in Fig. 1d), an increase in $\overline{u'v'}$ when $150\,\text {s}\le t\le 200\,\text {s}$ or $300\,\text {s}\le t\le 350\,\text {s}$ indicates the presence of the fire at or in the vicinity of this point (Fig. 8b). It can be seen from Fig. 1c–d that at this location, a fire-front that propagates south/east/southeast-ward spreads away from the IP. At $t=174$ s, 178.6  s, and 184 s, $u'(<0)$ and $v'(>0)$ (Fig. 8f,h) interact to impede the advancement of the the fire-front (peaks in Fig. 8d) at FP2. However, at $t=301$ s and $t=353$ s, the fluctuations $u'(>0)$ and $v'(<0)$ (Fig. 8f,h) interact to drive the fire southeast via bursts (peaks in Fig. 8d). Thus, increase in the magnitude of the horizontal momentum flux ($\overline{u'v'}$) and $u'v'$ is either symptomatic of fire-bursts that occur at irregular time intervals or representative of increased turbulence-induced impediment to the fire-spread; together, they play opposing roles in the fire-spread and in determining the spread rate.

### Length, time, and velocity scales

**Figure 9:**
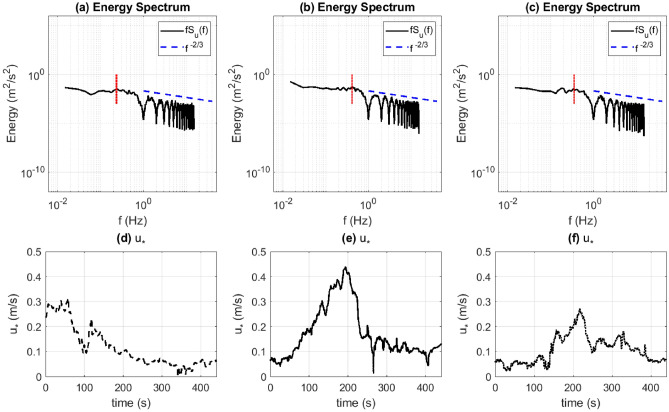
Energy spectrum (*fS*(*f*)) for the time-varying *u* signal at (**a**) the IP, (**b**) FP1, and (**c**) FP2, and $u_*$ computed at (**d**) the IP, (**e**) FP1, and (**f**) FP2. [Generated using MATLAB R2021a].

An understanding of the most dominant time and length scales can provide insights regarding the fluid dynamic mechanisms that assist or impede turbulent energy transport. Owing to the high sampling frequency of the velocity signals in this study, reliable estimates of the time scales associated with high-energy eddies can be obtained from the frequency spectrum. Figure 9 shows plots of *fS*(*f*) against *f* on a log-log scale, where *f* is the frequency and *S*(*f*) is the power spectral density computed using MATLAB’s *pwelch* function. Each plot in Fig. 9a–c represents the energy spectrum (*E*(*f*)) of the *u* velocity for a particular point in the flow field: the IP (Fig. 9a), FP1 (Fig. 9b), and FP2 (Fig. 9c). It is observed that the inertial sub-range for both the *u* spectrum (Fig. 9a–c) and *v* spectrum (not shown here) at all three points follows Kolmogorov’s $-2/3$ scaling law (since this is the spectral density pre-multiplied by the frequency) as shown by the blue dashed lines. Note that the energy spectrum does not capture the dissipation scales.

#### Integral length scale

The peak frequency ($f_p$) of the energy spectra for *u* at the three points mentioned above correspond to the most energetic eddies. This can be utilized to obtain the integral time scale ($\tau _u$) and integral length scale ($L_u$) as follows^49,50^:

2 $$\begin{aligned} \tau _u = \frac{1}{2\pi f_p}, ~ \kappa _p = \frac{2\pi f_p}{\overline{U}}, ~ L = \frac{1}{\kappa _p}. \end{aligned}$$

here $\kappa _p$ is the wave number of the most energetic eddies and $\overline{U}$ represents the largest absolute value of the moving mean $\overline{u}(t)$ for a given point. Table 1 summarizes the values of $f_p$, $\tau _u$, $\kappa _p$, and $L_u$ for the three points under consideration. From the table, the largest value of $L_u$ is 0.45 m. It is, therefore, evident that the most energetic eddies have wavelengths of approximately 0.45 m ($O(10^{-1})$ m) when measured along the *x* direction, which is approximately 1/5th of the domain length. The corresponding time scales range from 0.3  to  0.7 s ($O(10^{-1})$ s).

**Table 1:** A summary of the peak frequency and the corresponding time scale for the *u* energy spectrum, and the computed wave number and length scale for the IP, FP1, and FP2.

Point	$f_p$ (Hz)	$\overline{U}$ (m/s)	$\tau _u$ (s)	$\kappa$ (1/m)	$L_u$ (m)
IP	0.2344	0.62	0.6791	2.3871	0.419
FP1	0.410	1.16	0.3880	2.2237	0.450
FP2	0.352	0.42	0.4527	5.3023	0.189

#### Viscous dissipation scales

It must be noted that at the IP, the dissipation time scale ($\tau _\eta$) cannot be obtained from the energy spectrum (Fig. 9a). In order to obtain $\tau _\eta$, we first compute the friction velocity ($u_*$) from the 1-min averages of $u'w'$ and $v'w'$ as follows:

3 $$\begin{aligned} u_*^2 = (\overline{u'w'}^2 + \overline{v'w'}^2)^{1/2} \end{aligned}$$

The friction velocity computed from Eq. (3) is plotted for the IP, FP1, and FP2 in Fig. 9d–f. For a given point, the highest values of $u_*$ correspond to times of active combustion at that point. From a scaling perspective, we pick the largest value of $u_*$ from Fig. 9d–f, which is achieved at FP1 ($u_*= 0.44$ m/s in Fig. 9e). Assuming a first-order closure model with a gradient-diffusion parameterization, where the vertical turbulent shear stress can be parameterized by a turbulent diffusivity ($\nu _t$) and a gradient of the mean horizontal wind velocity (${\partial ||\overline{\mathbf {u}}||}/{\partial z}$)

4 $$\begin{aligned} u_*^2 = 0.44^2 = \nu _t \frac{\partial ||\overline{\mathbf {u}}||}{\partial z} \approx \nu _t \frac{\Delta ||\overline{\mathbf {u}}||}{\Delta z} \implies \nu _t \approx \frac{\Delta z}{\Delta ||\overline{\mathbf {u}}||}u_*^2. \end{aligned}$$

here $\nu _t$ is the eddy viscosity and “|| ||” represents computing the magnitude of a vector. We know that $\eta = \nu ^{3/4}\epsilon ^{-1/4}$ and $v_\eta = (\nu \epsilon )^{1/4}$, where $\eta$ represents the Kolmogorov length scale, $v_\eta$ is the Kolmogorov velocity scale, $\epsilon$ is the viscous dissipation rate of the TKE, and $\nu$ is the kinematic viscosity of air. From the *K*-$\epsilon$ turbulence model^51^:

5 $$\begin{aligned} \nu _t = C_{\mu } \frac{\bar{K}^2}{\epsilon } \implies \epsilon = C_{\mu }\frac{\bar{K}^2}{\nu _t}, \end{aligned}$$

where *K* represents the TKE and $C_\mu = 0.09$. From Eq. (4), we get:

6 $$\begin{aligned} \varepsilon = C_{\mu }\frac{\Delta ||\overline{\mathbf {u}}||\bar{K}^2}{\Delta z u_*^2} \approx 0.09\times \frac{1.3\times 0.4^2}{4.18\times 10^{-2}\times 0.44^2} = 2.34~ \text {m}^2/\text {s}^{3} \end{aligned}$$

here we have used $\Delta ||\overline{\mathbf {u}}||=1.3$ m/s and $\bar{K}\approx 0.4$  m^2^/s^2^ as computed at FP1 when $u_*$ reaches 0.44 m/s (Fig. 9e). The highest recorded surface temperatures are above 500^o^C (Fig. 3a–b). At 500^o^C, the kinematic viscosity of air ($\nu$) is approximately $7.8\times 10^{-5}$ m^2^/s^52^. This gives $\eta = 6.7\times 10^{-4}$ m and $v_\eta = 0.12$ m/s. Finally, the time-scale can be obtained using $\tau _\eta = \eta /v_\eta = 5.8\times 10^{-3}$ s. It is interesting to note that the value of $\tau _\eta$ is an order of magnitude lower than the sampling rate (1/30 s), which is why the energy spectrum (Fig. 9a–c) does not capture the viscous dissipation scales. The sampling frequency would have to exceed 100 Hz for that purpose. Furthermore, note that $v_\eta \sim 7.2$ m/min, which is an order of magnitude higher than the RoS (0.2 – 0.3 m/min) discussed in the next section.

To complete the discussion on length scales, we compute the Taylor microscale ($\lambda$) using the following equation^53^:

7 $$\begin{aligned} \lambda = \sqrt{\overline{u'^2}}\sqrt{15}\tau _\eta = 9.5\times 10^{-3}\,\text {m}. \end{aligned}$$

Here, we have used $\sqrt{\overline{u'^2}}\sim 0.425$ m/s as computed at FP1. Therefore, the Kolmogorov length scale is of $O(10^{-4})$, the Taylor microscale is of $O(10^{-3})$, and the integral length scale is of $O(10^{-1})$. Moreover, we report the Taylor Reynolds number to be $Re_\lambda = \frac{u'\lambda }{\nu } = 52$ and the turbulent Reynolds number for this study to be $Re_L = \frac{u'L}{\nu } = 2454$.

#### Rate of spread

**Figure 10:**
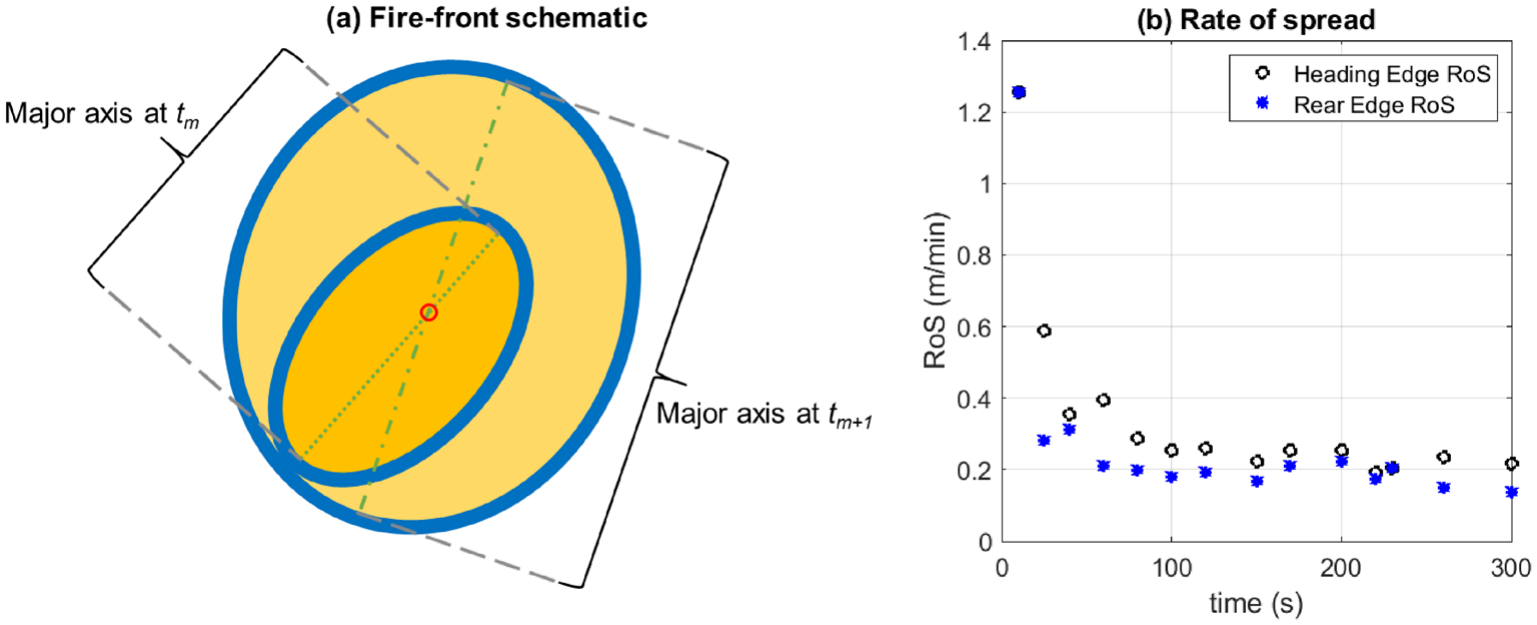
(**a**) A schematic diagram showing the major axis tracked at two instances of time elapsed since ignition to compute the corresponding distances of the heading and rear edges of the fire-front from the IP and (**b**) the variation of the heading and rear edge RoS with time. [MS PowerPoint 365 used to create (**a**); (**b**) generated using MATLAB R2021a].

An average empirical spread-rate for this fire can be obtained from a back-of-the-envelope calculation. From Fig. 1f, it can be seen that the fire-front traverses a distance of 1 m from the IP ($x=1.34$ m) to the eastward edge ($x=2.34$ m) in 350 s giving an average *x*-directional spread-rate of 0.17 m/min. The variation of the rate of spread with time is obtained as follows. A schematic of the location of the fire-front at two instances in time is shown in Fig. 10a. As discussed earlier, the fire-front is said to be located at the periphery of the updraft region (yellow) that shares its boundary with the downdraft region (dark blue) on the outside of it. At a given time ($t_m$ or $t_{m+1}$), the point on the fire-front furthest from the IP is said to be on the heading edge of the fire-front. The line joining this point to the IP gives the major axis of the ellipse (green dotted or dash-dotted lines), which intersects the ellipse again on the rear edge of the fire-front. The distances of these points from the IP at that this instant in time are used to calculate the rate of spread (RoS) of the heading and rear edges of the fire, estimates of which are shown in Fig. 10b. It can be seen that after an initial transient RoS up to about $t=50$ s, the RoS is relatively stable between 0.2 and 0.3 m/min. This is in close agreement with the average RoS calculated above and also suggests that the fire spreads very gradually in the absence of a preferred direction for the ambient wind. Moreover, the fire must overcome both, the effects of viscous dissipation and the resistance offered by the entrained air, in order to spread “outward”.

### Turbulent kinetic energy budget

**Figure 11:**
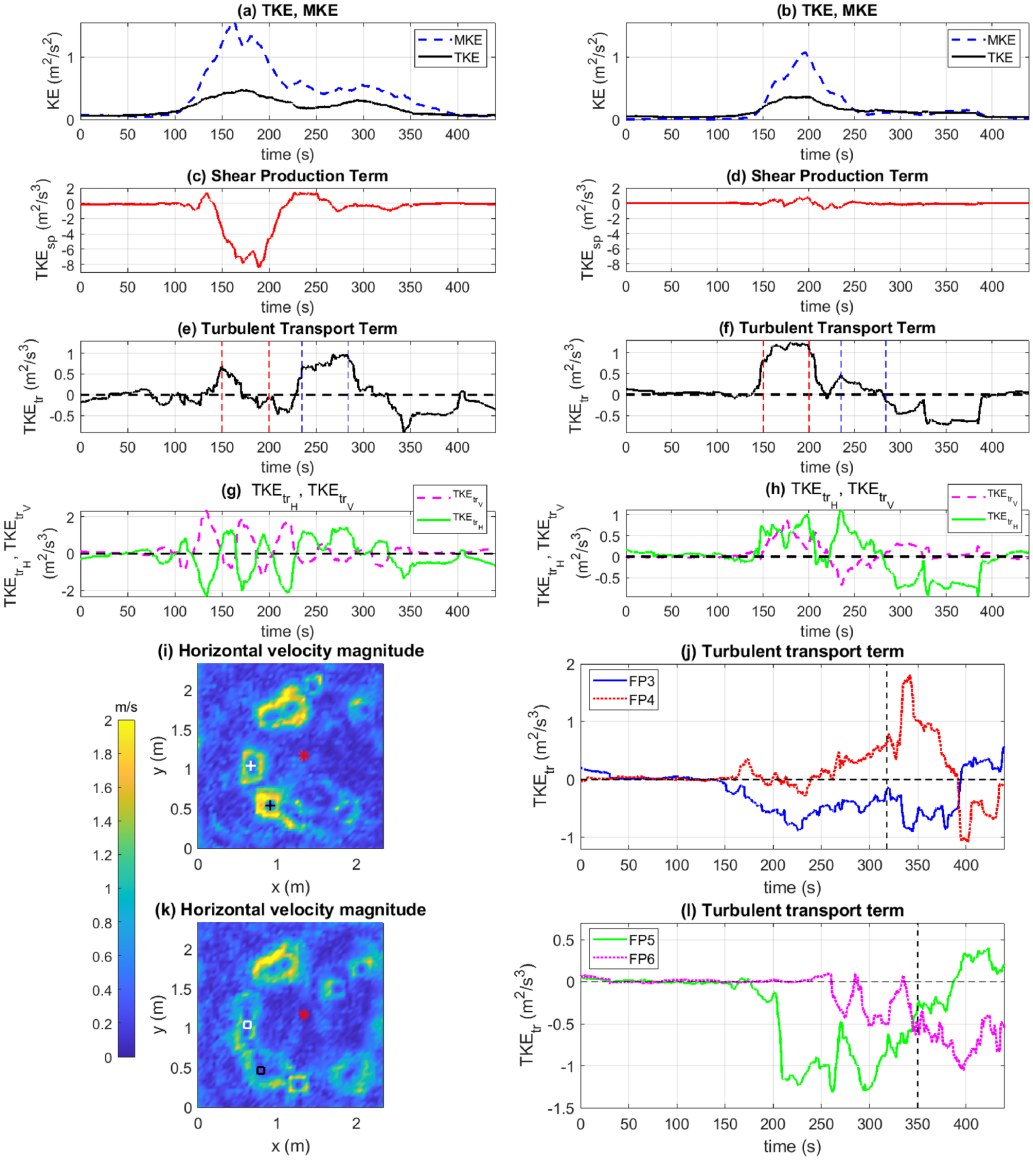
(**a**) TKE (black solid) and MKE (blue dashed), (**c**) $TKE_{sp}$, (**e**) $TKE_{tr}$, and (**g**) $TKE_{tr_V}$ (magenta dashed) and $TKE_{tr_H}$ (green solid) at FP1. (**b**) TKE (black solid) and MKE (blue dashed), (**d**) $TKE_{sp}$, (**f**) $TKE_{tr}$, and (**h**) $TKE_{tr_V}$ (magenta dashed) and $TKE_{tr_H}$ (green solid) at FP2. Vertical dashed lines in (**e**) and (**f**) correspond to $t=150$ s, 200 s, 235 s, and 284 s. Color contours of $u_H$ at $t=$ (**i**) 317.7 s (white cross represents FP3 and black cross represents FP4) and (**k**) 350 s (white square represents FP5 and black square represents FP6). $TKE_{tr}$ at (**j**) FP3 (blue solid line) and FP4 (red dotted line), and (**d**) FP5 (green solid line) and FP6 (magenta dotted line). [All panels generated using MATLAB R2021a].

We have seen that turbulent fluctuations play a role in accelerating or decelerating the fire spread via bursts or turbulence-induced impediment (eddies) at individual points on the fire-front. In this section, we look at the turbulent kinetic energy (TKE) and the terms in the TKE budget equation that contribute to the rate of change of TKE. In the past, studies have compared individual terms in the TKE budget equation with each other with a view to classify the flow based on the dominating mechanism^54^. The TKE budget equation is written as follows^55^:

8 $$\begin{aligned} \frac{\partial \overline{K}}{\partial t} + \bar{u}_j\frac{\partial \overline{K}}{\partial x_j}=\delta _{i3}\frac{g}{\theta _v}\overline{u'_i\theta '_v} - \overline{u'_i u'_j}\frac{\partial \bar{u}_i}{\partial x_j} - \frac{\partial \overline{u'_jK}}{\partial x_j} -\frac{1}{\rho }\frac{\partial \overline{u'_jp'}}{\partial x_j} -\epsilon , \end{aligned}$$

where *K* is the TKE, $\theta _v$ is the potential temperature (of air), and $p'$ is the pressure perturbation. The first, second, and third terms on the right hand side are the buoyant production ($TKE_{bp}$), shear production ($TKE_{sp}$), and turbulent transport terms ($TKE_{tr}$), respectively. While surface temperature measurements are available in this study, air temperatures are required to compute $TKE_{bp}$ and, therefore, the $TKE_{bp}$ term will be analyzed in a future study. In this section, we shall be exploring $TKE_{sp}$ and $TKE_{tr}$ for their role in the fire spread. The time-varying signals for each term will be compared at the two selected neighboring fixed points (FP1 and FP2) to get a deeper sense of the contribution of each term to the fire evolution. All partial derivatives referred to in this section are discretized using the procedure described in the Supplementary Information.

Figure 11a shows 1-min moving averages of the TKE ($\overline{K}$) and the mean kinetic energy (MKE, given by $(\overline{u}^2 + \overline{v}^2+ \overline{w}^2)/2$) computed at FP1, while $TKE_{sp}$ and $TKE_{tr}$ are shown in Fig. 11c,e, respectively. As is known from the literature^55^, TKE and MKE interact via the shear production term: $TKE_{sp}<0$ indicates a withdrawal of energy from the TKE and its addition to the MKE, while $TKE_{sp}>0$ indicates the contrary. It can be seen that the decrease in $TKE_{sp}$ when $140\,\text {s}\le t\le 220\,\text {s}$ (Fig. 11c) causes a loss in the TKE and a corresponding gain in the MKE (Fig. 11a). Since the mean flow at FP1 opposes the spread of the fire away from the IP in this time duration, the shear production term effectually helps resist the fire-spread at this point. At FP2, $TKE_{sp}\approx 0$ when $140\,\text {s}\le t\le 220\,\text {s}$ (Fig. 11d) indicating that the MKE does not increase much at the cost of the TKE. Therefore, the peak of the MKE (Fig. 11b) is much lower than that for FP1 (Fig. 11a). Relatively speaking, the shear production term does not contribute much in either assisting or resisting the fire spread at this point.

Now, let us examine the turbulent transport term ($TKE_{tr}$). For a closer examination, we divide it into its horizontal and vertical components:

9 $$\begin{aligned} TKE_{tr_H} = -\frac{\partial \overline{u'K}}{\partial x} -\frac{\partial \overline{v'K}}{\partial y}; ~~~~~ TKE_{tr_V} = -\frac{\partial \overline{w'K}}{\partial z}. \end{aligned}$$

As seen from Fig. 11, increased magnitudes of $TKE_{tr}$ at FP1 (Fig. 11e) are largely a consequence of increased magnitudes of $TKE_{tr_H}$, indicating that the contribution to $TKE_{tr}$ from $TKE_{tr_H}$ is much higher than the contribution of $TKE_{tr_V}$ (Fig. 11g). A similar argument can be made for FP2 (Fig. 11f, h)). An interesting feature of the flow is observed upon comparing the $TKE_{tr}$ signal at FP1 (Fig. 11e) with that at FP2 (Fig. 11f). The decreasing trend (and lower values) in $TKE_{tr}$ for $150\,\text {s}\le t\le 200\,\text {s}$ at FP1 concurs with the increasing trend (and higher values) observed at FP2. Conversely, for $235\,\text {s}\le t\le 284\,\text {s}$, the increasing trend (and higher values) of $TKE_{tr}$ at FP1 concurs with the decreasing trend (and lower values) at FP2. We have already seen that the horizontal TKE transport terms are the major contributors to $TKE_{tr}$. This suggests that TKE is exchanged between the two neighboring fixed points via a horizontal redistribution of TKE corresponding to the turbulent transport term.

The turbulent transport terms for two additional pairs of fixed points observed to have formed around $t=317.7$ s and $t=350$ s have been plotted in Fig. 11j,l, respectively. The western fixed point recorded at $t=317.7$ s is denoted by FP3 (white cross in Fig. 11i), while the eastern fixed point is denoted by FP4 (black cross in Fig. 11i). Similarly, the western fixed point recorded at $t=350$ s is denoted by FP5 (white square in Fig. 11k), while the eastern fixed point is denoted by FP6 (black square in Fig. 11k). At FP3 and FP4, trends in $TKE_{tr}$ are similar until $t=317.7$ s after which increasing trends of $TKE_{tr}$ at FP4 concur with decreasing trends at FP3 (Fig. 11j). Similarly, increasing trends of $TKE_{tr}$ at FP5 concur with decreasing trends of $TKE_{tr}$ at FP6 as seen from Fig. 11l after $t=350$ s suggesting the possibility of TKE exchange between these two fixed points via the turbulent transport terms.

The analysis above has implications for kinematic fire-growth models based on Huygen’s principle^6,8^. While such models have provided a useful framework for predicting the shape and location of the fire perimeter at a given time from ignition, not much was known about the interaction of fires along the fire-front. The analysis above suggests that adjacent local fires can interact via the turbulent transport terms. Moreover, the horizontal turbulent flux at each point on the fire perimeter plays a significant role in the fire spread at a given time during its evolution, as seen above.

## Discussion

**Figure 12:**
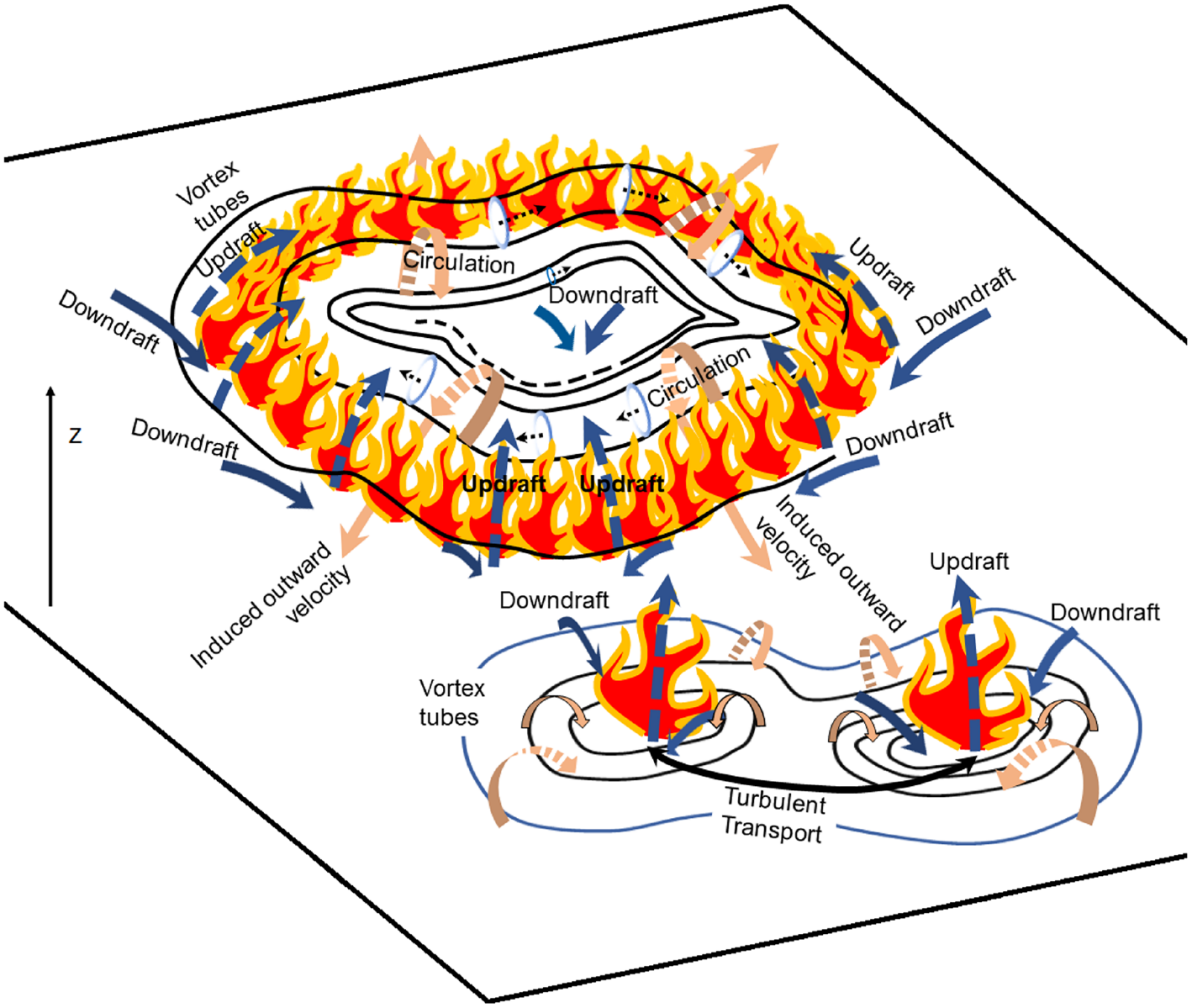
A brief illustration of the (pre- and post- fixed point formation) flow dynamics in the presence of the flame. Figure is not to scale. [Created in MS PowerPoint 365].

In this paper, we have analyzed quasi-two-dimensional PIV data sampled at a high frequency (30 Hz) during a laboratory-scale burn experiment in a relatively small domain ($2.34\,\text {m}\times 2.34\,$m) under calm ambient wind conditions (and the lack of a preferential wind-forcing direction). The turbulent Reynolds number is reported to be approximately 2454. Observations from this study are illustrated briefly in Fig. 12. Horizontal velocity vectors and thermal data captured by the cameras show that the fire (or more appropriately, the hot flame) draws cool air from the surrounding region (entrainment). This local wind exerts a dynamic pressure upon the fire-front, thereby offering resistance to the fire spread. Moreover, streamlines penetrating the fire-front get curved away from the fire-core, meet near the inner edge of the fire-front, and appear to shield the fire perimeter from the innermost core of the fire. Again, the streamlines do not carry signatures of wind-driven fire spread. Rather, the fire seems to be propelled by the radially outward velocity induced close to the surface of the fuel bed by the vortices of neighboring vortex tubes. These vortex tubes, which originate on the outside of the fire and spiral inward, get stretched thinner at the location of the fire-front before dispersing again at the fire core. Correspondingly, the horizontal vorticity magnitude increases strongly at the fire perimeter, and then reduces near the fire core where the vorticity eventually dissipates. Moreover, circulation structures obtained from the horizontal vorticity vectors confirm the presence of counter-rotating vortex pairs on opposite sides reported elsewhere as a key mechanism for fire spread^34^. While Finney et al.^34^ observed these vortex pairs from images of laboratory fires, the present experiment enables us to quantify these vortices for the first time, which is a significant contribution of this study.

Additionally, vortices that comprise the vortex tubes, precess alternately toward and away from the surface of the fuel bed, causing the vortex tubes to be kinked. The strong updraft observed at the location of the fire-front would advect the kinked vortex tubes vertically upward. In the presence of the active vertical vorticity component observed here or in the event of tilting of the vortex tubes, this can result in a fire whirl (albeit weaker than whirls observed in large-scale fires). Moreover, the presence of vorticity and radial entrainment are known to provide conditions that are conducive to the formation of fire whirls^48^.

The integral length scale obtained from the spectral analysis suggests that the most energetic eddies have wavelengths of approximately a fifth of the domain length ($O(10^{-1})$ m) when measured along the *x* direction. In addition, increase in the magnitude of turbulent fluxes of horizontal momentum signal the presence of either fire bursts that assist the fire spread or fire-spread-impeding horizontal eddies. The overall consequence of the processes at play (including radiative heat transfer) is that the fire spread is relatively gradual, with a RoS of approximately 0.2–0.3 m/min. Time-dependent RoS values have also been obtained, which can be used as geometric parameters in well-documented kinematic models such as the first-order growth models of Richards^6–8^.

The structure of the fire also alters in response to its increasing perimeter and the local wind conditions. Cross-correlation contours of the vertical velocity clearly show the elliptical shape of the fire perimeter in the early stages of fire-growth along with its re-orientation in response to change in the local wind conditions or fuel-bed heterogeneity. As the fire progresses, the unstable fire-front disintegrates into smaller pockets of fire. The horizontal flow around these pockets is similar to the behavior of a chaotic system in the vicinity of an attractor. In such a case, the envelope of these fire pockets constitutes the fire-front. The envelope of fire-fronts expands as the pockets relocate further away from the IP. This behaviour suggests that the wave propagation model of Richards^6^ (based on Huygen’s principle) is a reasonable model for fire growth. However, such kinematic models are unable to predict the response of the fire-front to instabilities, which can be clearly observed from the data analyzed in this study.

While Huygen’s principle of wave propagation has been the basis of several fire-growth models, not much is known about how the local ellipses are affected by local changes in the flow. The advantage of decomposing the flow into its mean and turbulent components is that the contribution of the turbulent fluctuations in assisting or resisting fire-growth via bursts or fire-spread-impeding eddies, respectively, can be analyzed. Moreover, not much has been documented about the mechanism of energy exchange between neighboring (local) ellipses. This study presents the turbulent transport term of the TKE budget equation as a possible source of energy exchange between such ellipses. Historically, it is known that nearby fires are attracted to each other. As seen from the horizontal velocity contours, neighboring fire pockets in this study are also found to coalesce along the fire-front. The interaction between two nearby fires via the turbulent transport term provides a possible explanation for this.

At this point, we would like to address some of the limitations associated with this work. Although the wind data from a local weather station (Whitehall Forest) suggests calm ambient wind conditions, the absence of real-time ambient wind measurements makes it difficult to track the exact wind shifts that could explain the north-eastern predilection of the fire at later times during the experiment (as observed in Fig. 1d–f). Typically, the high variability in the local wind conditions during a burn experiment conducted in the open and the sensitivity of the fire-induced turbulence to these conditions makes it difficult to reproduce the results of such experiments exactly^42^. Furthermore, considering that the pine litter used in this experiment was hand drawn, we also expect some heterogeneity in the fuel distribution, which could explain the north-eastern predilection of the fire. In addition, the vertical component of velocity is obtained by using zero divergence in the velocity field and is available only at one height above the surface of the fuel bed. The absence of vertical resolution for this data, i.e. the (quasi-) two-dimensional nature of the measurements makes it difficult to establish the presence of fire whirls more concretely.

Despite its limitations, this experiment was unique in its ability to collect high frequency PIV data during a burn experiment, notwithstanding the challenges associated with measuring the velocity field in the presence of a flame^42^. The ability to resolve both in time (with a high frequency) and in space has allowed us to quantify some of the canonical features reported previously by different studies of fire behavior in small-scale surface fires (such as the influx of ambient air into the fire core^42,43^ , the acceleration of the wind across the fire-front^42,43^, and the presence of counter-rotating eddies^34^) and find additional fire behavior trends that have not been reported elsewhere. Therefore, from this perspective, we have been able to “reproduce” some of the canonical features reported in previous studies, while also contributing additional insights. Moreover, this study is able to provide insights into fire behavior while circumventing the issues associated with damage to equipment or personnel life during large-scale field experiments or prescribed burns.

We have seen that upon the onset of instability, the fire disintegrates into smaller pockets concentrated around fixed points. In a subsequent study, a nonlinear bifurcation analysis shall be conducted to investigate the stability behaviour of a grassland fire where the fixed points can be treated as chaotic attractors. After remaining relatively static for a certain amount of time, the fixed points relocate further away from the ignition point as discussed above. In time, neighboring fixed points coalesce leading to larger pockets of fire concentrated at a locus of fixed points. These can be treated as different modes of post-instability behaviour. The results of this analytical work can be compared with the fire behaviour documented above. Furthermore, owing to the need for repeatability, the authors are planning on conducting a similar laboratory-scale burn experiment in the open, in the near future. Moreover, a laboratory-scale burn experiment with three-dimensional data collection would facilitate further analysis of potential fire whirls and is currently anticipated. Since fire-fighters train to track fire behavior from one moment to the next, we hope that this work will assist them in accomplishing the same via a broader understanding of fire dynamics.

## Methods

**Figure 13:**
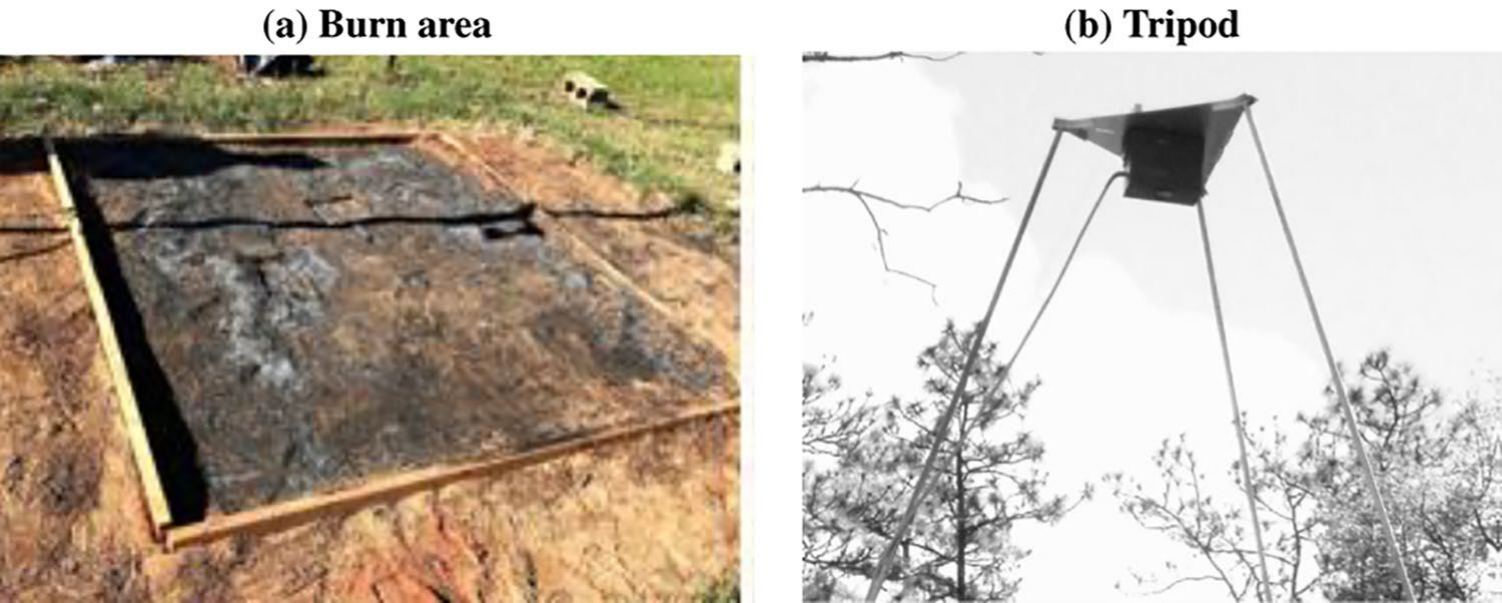
(**a**) Post burn photograph of burn area and (**b**) the tripod housing the FLIR SC660 and GoPRO HERO3 cameras.

The data analyzed in this study were collected during a burn experiment conducted by the US Forest Service, Southern Research Station (Savannah, Georgia, USA). A 4 m$\times$ 4 m sand bed was constructed using all-purpose construction sand at the Whitehall Forest on the campus of the University of Georgia, in Athens. A 2.34 m $\times$ 2.34 m burn area was established inside the sand bed, marked by a wooden frame (Fig. 13a). Hand-spread pine needles mimicking natural needle cast in field situations constituted the fuel. The fuel loading was approximately 370 g/m^2^, which was similar to fine fuel loading measured in a longleaf pine stand with a fire return interval of 1–2 years^56^. Fuel moisture content was 4 %. Grid points were spaced 4.18 cm apart in both the latitudinal (*x*) and longitudinal (*y*) directions. The fire was ignited at a point given by $x=1.34$ m and $y=1.17$ m and allowed to spread without any intentional external human interference.

The imaging system consisted of a 7 m-tall aluminum tripod with a FLIR (Forward Looking Infrared) SC660 (FLIR Systems Inc., Boston, MA, USA) thermal imaging system positioned directly above burn area to provide a nadir view (Fig. 13b). The FLIR system has a focal plane array of 640 $\times$ 480 pixels, a spatial resolution of 1.3 mrad, a sensitivity of 0.03 °C, and a thermal accuracy of ± 2%. The temperature range selected for data collection during the fire was 100–650 °C at a measurement rate of 1 Hz. Further details on FLIR specifications are found in the literature^57–59^. Visual imagery was captured by a GoPRO HERO3 camera collocated with the FLIR. Resolution of the video imagery is 1920 $\times$ 1080 pixels and was captured at a frame rate of 30 fps.

The flow field in the vicinity of the fire was estimated by applying cross-correlation particle image velocimetry (PIV). Our PIV implementation was inspired by the work of Fujita and Hino^60^, who used an unseeded PIV method to estimate river flows. In their work, Fujita and Hino had intended to use the unseeded PIV measurement technique for small ripples, resulting from wall-bounded turbulence, on the water surface. Instead, they used the method for large-scale patterns, caused by the interaction of boil vortices generated near the river bed with the water surface, which typically occurs in high river-flow conditions. This choice was a consequence of video images being taken from a helicopter, which made it difficult to capture small ripples on the water surface. In the current study, the unseeded PIV measurement technique could be applied in slow-flow conditions since the camera was located relatively close to the fuel bed, i.e. at a height of 7 m. While the methodology of Fujita and Hino relied on patterns generated by the interaction of boil vortices with the water surface as natural unseeded tracers, we have relied on patterns generated by the fire-flames, smoke, and ash particles as our unseeded tracers. Next, the cross-correlation PIV was implemented in Python using the openpiv module (Python version 3.8.5 and Anaconda environment version 4.9.2). The interrogation window was set at 24 pixels with a window overlap of 12 pixels and a search-area size set as 2.5 times the interrogation window size. The video from the GoPRO camera was trimmed to a 2.34 m $\times$ 2.34 m area in the center of the burn area for an image size of 888 $\times$ 888 pixels, and split into sequential images with a time step of 1/30 of a second.

Velocity data were collected for approximately 440 s ($t_T$) and the sampling frequency was 30 Hz (as mentioned above). Two components of velocity were measured: the latitudinal (*x*) component denoted by *u* and the longitudinal (*y*) component denoted by *v*. Here, $u>0$ when the latitudinal flow is eastward, i.e. in the $+x$ direction and $v>0$ when the longitudinal component is northward, i.e. in the $+y$ direction. The vertical velocity (*w*) was obtained from mass conservation: the divergence of the net velocity vector ($\nabla .\mathbf {u}$) was set to zero and the resulting equation was integrated to a height equal to the cell-size in the horizontal domain (4.18 cm) as shown in the Supplementary Information. No penetration conditions were enforced at the surface, i.e. $w|_{z=0} = w_0 = 0$. We assumed that the velocity measurements were made at a height $z = 4.18$ cm from the surface ($\Delta z = 4.18$ cm). The vertical velocity component (*w*) so computed is said to be positive ($w>0$) if directed opposite to the gravitational force.

The burn experiment was conducted during the afternoon of November 15, 2017. The weather station located in Whitehall Forest recorded mean wind speeds of 0.6 and 0.5 m/s for the hours of 1700 and 1800 UTC, respectively, with the mean direction varying between 77° and 152° during these hours. The maximum gust recorded during these hours was 1.21 m/s. These wind speeds were measured at a standard height of 10 m above ground level (AGL) and were adjusted to obtain the wind speeds at a height of 1 m AGL by fitting a logarithmic profile. The strongest gust measured at the height of 10 m corresponds to a speed less than 0.3 m/s at a height of 1 m. Similarly, mean wind speeds at 1 m were calculated to be less than 0.16 m/s. According to their meteorological definition, calm winds are said to have speeds that are less than 0.5 m/s or 1 knot. Since the wind conditions fulfilled this criterion near the burn site, it was expected that the wind forcing would be minimal, especially close to the surface of the fuel bed. Therefore, ambient wind data were not collected for this site during the experiment. A possible alternative for ambient wind measurements has been discussed in the Supplementary Information.

Vorticity estimates were obtained at a height $z=4.18$ cm from the surface by taking the curl of the velocity vector $(\omega _i = \epsilon _{ijk}\frac{\partial u_k}{\partial x_j}~\text {in indicial notation})$ and using the procedure described in the Supplementary Information to discretize the partial derivatives. No-slip boundary conditions were imposed at the surface ($u|_{z=0}=v|_{z=0}=0$).

A Reynolds decomposition was applied on the horizontal and vertical velocity components ($u_i = \overline{u}_i + u_i'$, where $i=1,2,3$). The mean parameters ($\overline{u}_i$) were computed from 1-min moving averages over the time-varying signal at each point in the domain. These were utilized to compute the mean kinetic energy (MKE), given by $(\overline{u}^2 + \overline{v}^2+ \overline{w}^2)/2$, at selected neighboring points in the flow field. The fluctuating parameters ($u_i'$) were used to compute the turbulent momentum fluxes in the horizontal plane ($\overline{u'v'}$) and vertical plane ($\overline{u'w'}$, $\overline{v'w'}$) at these points. The 1-min moving averages of the TKE, given by $\overline{K} = (\overline{u'^2} + \overline{v'^2}+ \overline{w'^2})/2$, were also obtained. The transport and shear production terms of the TKE budget equation are analyzed at these points for insights into the transaction of TKE between them.

## Supplementary Information

Supplementary Information.

## Data Availability

All data used in the analysis shall be made available upon request to the Southern Research Station, US Forest Service. All codes used in the analysis shall be made available upon contacting the authors at the University of California, Irvine.
