# Supplementary material for: Investigating the turbulent dynamics of small-scale surface fires
Source: Sci Rep. 2022 Jun 22;12:10503. doi: 10.1038/s41598-022-13226-w (PMC9217930; doi:10.1038/s41598-022-13226-w)
Supplement: Supplementary file 1 — Supplementary Information. [file 41598_2022_13226_MOESM1_ESM.pdf]

Investigating the turbulent dynamics of small-scale surface fires

Ajinkya Desai[1,*], Scott Goodrick[2], and Tirtha Banerjee[1]

[1]Department of Civil and Environmental Engineering, University of California, Irvine, CA 92697, USA
[2]USDA Forest Service, Southern Research Station, Athens, GA 30602, USA

Supplementary Information

Discretization Schemes

In this section, we describe the procedure to discretize all spatial partial derivatives referred to in this study. Let F represent either u, v, or w, or their respective mean or fluctuating parts. A central difference scheme is used to discretize partial derivatives in the x and y directions:

$$\left.\frac{\partial F}{\partial x}\right|_{z=\Delta z} = \left.\frac{F_{i+1,j} - F_{i-1,j}}{2\Delta x}\right|_{z=\Delta z}, \; \left.\frac{\partial F}{\partial y}\right|_{z=\Delta z} = \left.\frac{F_{i,j+1} - F_{i,j-1}}{2\Delta y}\right|_{z=\Delta z}. \tag{S1}$$

Here, $\Delta x = \Delta y$; they represent the horizontal cell-size of the domain. A backward difference scheme is used for partial derivatives in the z direction:

$$\left.\frac{\partial F}{\partial z}\right|_{z=\Delta z} = \frac{F_{i,j}|_{z=\Delta z} - F_{i,j}|_{z=0}}{\Delta z}. \tag{S2}$$

No slip and no penetration boundary conditions imply that $F_{i,j}|_{z=0} \equiv 0$. Equations (S1) and (S2) are used to derive w as follows:

$$\frac{\partial u}{\partial x} + \frac{\partial v}{\partial y} + \frac{\partial w}{\partial z} = 0; \implies w|_{z=\Delta z} = w_0 - \Delta z \left[\frac{u_{i+1,j} - u_{i-1,j}}{2\Delta x} + \frac{v_{i,j+1} - u_{i,j-1}}{2\Delta y}\right]. \tag{S3}$$

The three components of vorticity $(\omega_x,\ \omega_y,\ \omega_z)$ at $z = \Delta z$ are obtained using Eqs. (S1) and (S2) as follows:

$$\begin{aligned} \omega_x &= \frac{w_{i,j+1} - w_{i,j-1}}{2\Delta y} - \frac{v_{i,j} - v|_{z=0}}{\Delta z}, \; \omega_y = \frac{u_{i,j} - u|_{z=0}}{\Delta z} - \frac{w_{i+1,j} - w_{i-1,j}}{2\Delta x}, \\ \omega_z &= \frac{v_{i+1,j} - v_{i-1,j}}{2\Delta x} - \frac{u_{i,j+1} - u_{i,j-1}}{2\Delta y}. \end{aligned} \tag{S4}$$

The shear production term of the TKE budget equation (TKE_{sp}) is first written out in full:

$$
\begin{aligned}
TKE_{sp} = -\overline{u'v'}\left(\frac{\partial \bar{u}}{\partial y} + \frac{\partial \bar{v}}{\partial x}\right) - \overline{u'w'}\left(\frac{\partial \bar{u}}{\partial z} + \frac{\partial \bar{w}}{\partial x}\right) - \overline{v'w'}\left(\frac{\partial \bar{v}}{\partial z} + \frac{\partial \bar{w}}{\partial y}\right) \\
-\overline{u'^2}\left(\frac{\partial \bar{u}}{\partial x}\right) - \overline{v'^2}\left(\frac{\partial \bar{v}}{\partial y}\right) - \overline{w'^2}\left(\frac{\partial \bar{w}}{\partial z}\right).
\end{aligned}
\tag{S5}
$$

Each term is then discretized at a grid point given by grid number (i, j) using Eqs. (S1) and (S2). Finally, the horizontal and vertical parts of the turbulent transport term (TKE_{tr_H} and TKE_{tr_V}, respectively) are obtained using Eqs. (S1) and (S2) as follows:

$$
\begin{aligned}
TKE_{tr_H} &= -\frac{\partial \overline{u'K}}{\partial x} - \frac{\partial \overline{v'K}}{\partial y} = \frac{\overline{u'_{i-1,j}K_{i-1,j}} - \overline{u'_{i+1,j}K_{i+1,j}}}{2\Delta x} + \frac{\overline{v'_{i,j-1}K_{i,j-1}} - \overline{v'_{i,j+1}K_{i,j+1}}}{2\Delta y}, \\
TKE_{tr_V} &= -\frac{\partial \overline{w'K}}{\partial z} = -\frac{\overline{w'_{i,j}K_{i,j}}|_{z=\Delta z} - \overline{w'_{i,j}K_{i,j}}|_{z=0}}{\Delta z} = -\frac{\overline{w'_{i,j}K_{i,j}}|_{z=\Delta z} - 0}{\Delta z}.
\end{aligned}
\tag{S6}
$$

Wind Velocity Vectors

In the absence of ambient wind data, we analyze the horizontal velocity vectors at the domain edges as the next approximation. The normalized horizontal velocity vectors at the northern, southern, western, and eastern edges of the domain have been computed and plotted in Fig. S1 against non-dimensionalized time (t/t_T). The horizontal velocity at a domain edge, at a given time, is computed from a spatial average of the horizontal velocity at all the points lying on that edge. It is denoted by $\mathbf{u}_{HF} = u_F\hat{\mathbf{i}} + v_F\hat{\mathbf{j}}$ in Fig. S1, where $F = N, S, W,$ or E; the letters represent the northern, southern, western, or eastern edge of the domain, respectively. All vectors are normalized by the magnitude of the longest wind vector from among all edges ($||\mathbf{u}_{HF,max}||$, denoted simply by $u_{HF,max}$).

It is important to comment on the north-eastward penchant of the fire spread, in the absence of a specific ambient wind-forcing direction, as seen from Fig. 1. From Fig. S1, it is observed that the normalized $\mathbf{u}_{HN}$ is predominantly southward and much stronger (Fig. S1(b)) than the normalized $\mathbf{u}_{HS}$, which is predominantly northward (Fig. S1(d)). Furthermore, the strength of the normalized $\mathbf{u}_{HS}$ only increases towards the end of the experiment ($t/t_T > 0.7$). This is symptomatic of the preference of the fire to spread more towards the north compared to the south, since the ambient in-drafts are stronger closer to the flame. Similarly, the normalized $\mathbf{u}_{HE}$ is predominantly westward and stronger (Fig. S1(c)) than the normalized $\mathbf{u}_{HW}$, which is predominantly eastward (Fig. S1(a)). Again, this is symptomatic of the preference of the fire spread towards the east. Overall, these are signatures of the fire spread towards the north-east (Fig. 1(d)-(f)). Therefore, it is important to note that while horizontal velocity vectors computed at the domain edges are suggestive of the response of the ambient air to the presence of the flame, they are not to be interpreted as wind forcing for the fire spread. Considering the lack of a specific

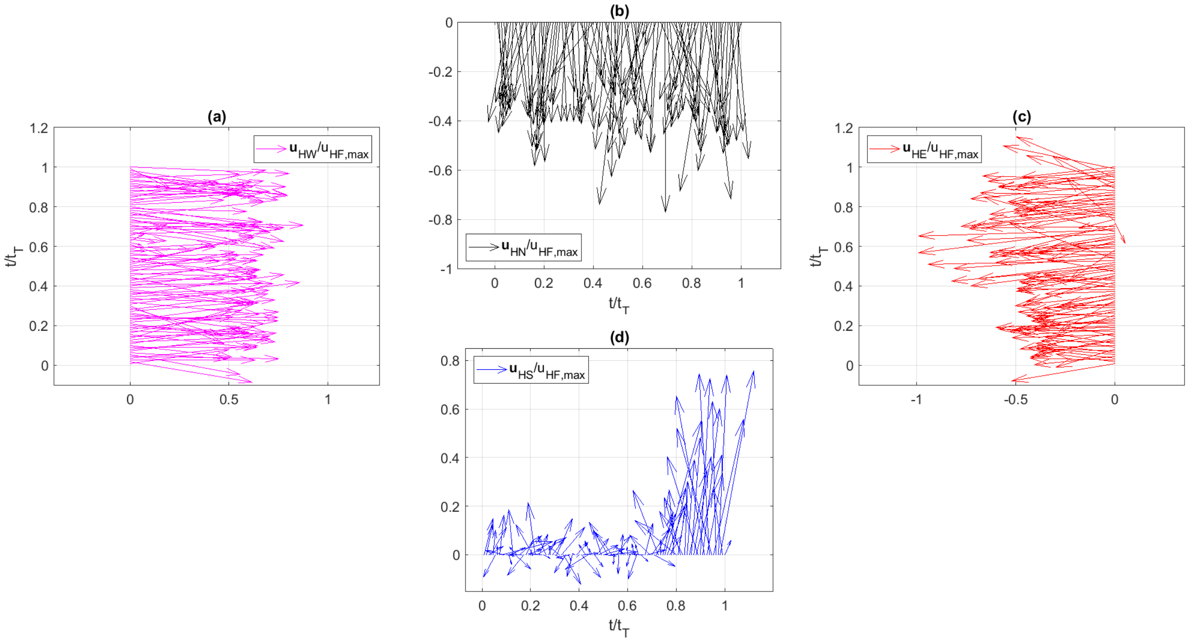

Figure S1: Normalized wind vectors at the (a) West, (b) North, (c) East, and (d) South Faces of the domain plotted against time non-dimensionalized by the total time ($t_T = 440$ s). The same scales are shared by (a) and (c), on one hand, and (b) and (d), on the other.

ambient wind direction in this experiment, the inclination of the fire to spread towards the north-east can likely be explained by possible heterogeneity in the fuel bed and shifts in the local wind.
